# Enhancement of Arabidopsis growth characteristics using genome interrogation with artificial transcription factors

**DOI:** 10.1371/journal.pone.0174236

**Published:** 2017-03-30

**Authors:** Niels van Tol, Martijn Rolloos, Johan E. Pinas, Christiaan V. Henkel, Dieuwertje Augustijn, Paul J. J. Hooykaas, Bert J. van der Zaal

**Affiliations:** 1 Institute of Biology Leiden, Faculty of Science, Leiden University, Leiden, The Netherlands; 2 BioSolar Cells, Wageningen, The Netherlands; 3 Leiden Institute of Chemistry, Faculty of Science, Leiden University, Leiden, The Netherlands; Instituto de Biologia Molecular y Celular de Plantas, SPAIN

## Abstract

The rapidly growing world population has a greatly increasing demand for plant biomass, thus creating a great interest in the development of methods to enhance the growth and biomass accumulation of crop species. In this study, we used zinc finger artificial transcription factor (ZF-ATF)-mediated genome interrogation to manipulate the growth characteristics and biomass of Arabidopsis plants. We describe the construction of two collections of Arabidopsis lines expressing fusions of three zinc fingers (3F) to the transcriptional repressor motif EAR (3F-EAR) or the transcriptional activator VP16 (3F-VP16), and the characterization of their growth characteristics. In total, six different 3F-ATF lines with a consistent increase in rosette surface area (RSA) of up to 55% were isolated. For two lines we demonstrated that 3F-ATF constructs function as dominant *in trans* acting causative agents for an increase in RSA and biomass, and for five larger plant lines we have investigated 3F-ATF induced transcriptomic changes. Our results indicate that genome interrogation can be used as a powerful tool for the manipulation of plant growth and biomass and that it might supply novel cues for the discovery of genes and pathways involved in these properties.

## Introduction

Substantial increases in the yield of many important crop species have been achieved over the last couple of decades [1], but for a number of different crop species there has been a stagnation in the improvement of yield since the late 1990s [1–3], indicating that breeders have to a large extent exhausted the existing tools for yield improvement. Currently, photosynthetic efficiency is regarded as one of the primary targets for the further enhancement of crop yield [1, 4]. As the overall solar energy to biomass conversion efficiency of photosynthesis is generally regarded as low [1, 5], the improvement of photosynthetic efficiency is expected to allow for substantial yield increases. Therefore, there is great interest in the development of novel tools and breeding techniques.

The plant species *Arabidopsis thaliana* has been used as a model system for the discovery of genes that are involved in growth and the accumulation of biomass [6]. The mechanisms of rosette development and growth of Arabidopsis plants have been well documented [7], and ectopic overexpression or mutation of a substantial number of so-called Intrinsic Yield Genes (IYGs) has been demonstrated to enhance the growth of Arabidopsis through various mechanisms involving the rates of cell expansion and cell division [6]. In some cases IYGs were also able to confer an increase in the growth of other plant species [8–10]. There is also variation in growth rate among natural Arabidopsis accessions, many of them being much larger than the most commonly studied wild-type accession Columbia-0 (Col-0). Through the discovery of hybrid vigor among crosses between accessions the (epi)genetic factors leading to growth and biomass increases are being established [11–14]. As is the case for Arabidopsis, enhancement of crop yield has mostly been achieved in single (mutant) genotypes or crosses between genotypes. These classical strategies have had a great impact on yield, but are being exhausted and will most likely not be sufficient to meet the future demands as described above.

Using Arabidopsis as a model system, we have investigated whether the artificial distortion of gene expression patterns as a novel type of epigenetic variation can trigger plants to develop rosettes with a larger surface area and to accumulate more biomass. A promising method to do so has been coined ‘genome interrogation’ and is originally based on the use of zinc finger artificial transcription factors (ZF-ATFs) to drastically change genome-wide transcription patterns and to induce novel phenotypes of interest [15]. While initially successfully applied in unicellular organisms or cell cultures [16–18], studies in our lab have demonstrated that genome interrogation is also possible in plants [19–21]. In our setup, arrays of three zinc fingers (3F) are used, which each recognize a cognate 3 base pair (bp) DNA consensus sequence of 5’-GNN-3’ [22], with N being any of the four bases. From the sixteen 5’-GNN-3’ binding ZFs, 4096 3F combinations can be generated that each function as DNA binding domains recognizing a 9 bp target sequence on average occurring once in approximately 130,000 bp of genomic DNA. When fused to an effector protein domain that is known to influence transcription of genes, ZF-ATFs can address a plethora of genomic loci simultaneously for differential expression leading to a phenotype of interest in a multicellular organism. Even in the relatively small genome of Arabidopsis each particular 9 bp sequence will on average occur approximately 1000 times. The level and potential tissue specificity of ZF-ATF expression in the organism of interest depends on the choice of the promoter controlling the transgene. In Arabidopsis the promoter of the *RPS5a* gene, which is predominantly active in embryonic and meristematic tissue [23], proved to be suitable for genome interrogation experiments with ZF-ATFs [19]. In previous studies conducted in our lab aimed at finding novel mutants with enhanced somatic recombination [19] and salinity tolerance [21], we have successfully used an effector domain originating from the VP16 protein of the herpes simplex virus as a potent transcriptional activator [24]. In the present study, we also explored the possibilities for genome interrogation in Arabidopsis using the ERF-associated Amphiphilic Repression (EAR) motif, which originates from Arabidopsis [25, 26] and has been reported to confer dominant repressor activity on transcription factors [27].

Here, we describe the construction and phenotypic characterization of two collections of Arabidopsis genome interrogation lines harboring 3F-EAR encoding T-DNA constructs (~700 lines) and 3F-VP16 encoding T-DNA constructs (~4200 lines). A multitude of different phenotypes was found in the 3F-EAR collection, and some of the plants were substantially larger than the Col-0. Although the increase in growth of these lines is likely to have been caused by insertion and expression of a 3F-EAR encoding T-DNA construct, transformation of Col-0 plants with reconstituted T-DNA constructs encoding recovered 3F-EAR fusions did not prove a causal connection with enhanced growth. However, in the larger 3F-VP16 collection we found lines for which we were also able to prove that the 3F-VP16 expression constructs are indeed causative agents for strong increases in surface area and biomass. We further describe 3F-ATF induced transcriptomic changes that might be indicative for increased growth and biomass in Arabidopsis. Our results not only suggest that genome interrogation is a powerful tool for the manipulation of the growth and the transcriptome of Arabidopsis, but also that it can be regarded as a novel strategy to discover genes and pathways for the improvement of yield in plants.

## Materials and methods

### Growth conditions and plant material

All plants were grown on a 9:1 (vol:vol) mixture of potting soil (Horticoop B.V., Bleiswijk, The Netherlands) and sterile laboratory sand (0.3 mm—0.6 mm grains; Het Noorden B.V., Nieuwe Pekela, The Netherlands) in a climate-controlled growth chamber at a constant temperature of 20°C, 70% relative humidity, at a light intensity of approximately 200 μmol m^-2^ s^-1^ of photosynthetically active radiation (PAR) generated with metal halide lamps (400 W, type HSI-TSX Britelux, Sylvania) at a 12 h photoperiod. The Arabidopsis accession Columbia-0 (Col-0) was used as wild-type and as the background genotype for all transformations.

### Construction of the binary vector pRF-EAR-Kana and the collection of 3F-EAR expressing Arabidopsis lines

The amino acid sequence of the EAR (SRDX) domain (LDLDLELRLGFA) was derived from Hiratsu *et al*. [27]. A double stranded oligonucleotide encoding this amino acid sequence (5’-GGTACCGAGGCCCAGGCGGCCTCGAGAACTAGTGGCCAGGCCGGCCAATTGGATTTGGATTTGGAATTGAGATTGGGATTTGCTTAGGAGCTC-3’; codon-optimized for Arabidopsis) with *Sfi*I sites for cloning of 3F domains was generated in a previous study [19] and cloned into *Kpn*I and *Sac*I digested pSDM3835 [28]. The resulting binary vector construct, designated pRF-EAR-Kana, allows for expression of 3F domains preceded by an N-terminal FLAG-tag and an SV40 derived nuclear localization signal (NLS), fused to the C-terminal EAR domain under control of the promoter of the Arabidopsis *RPS5a* gene. The same procedure was followed for plasmid pSDM3838 [28], resulting in an identical coding region under control of the constitutive CaMV *35S* promoter, and was designated p35S-EAR-Kana. 3F encoding sequences from subpools 1, 5, 7, 9, 11, 13, and 15 [19] were used for 3F-EAR vector library construction with more than 200 clones per pool in *E*. *coli*, apart from pools 7 and 13, which only contained about 100 clones. Each subpool of pRF-EAR-Kana plasmids encoding 3F-EAR fusions was mobilized into to the Agrobacterium strain Agl1 and was used to generate transgenic Arabidopsis plants in Col-0 background as described previously [19].

### Rosette surface area analysis of the collection of 3F-EAR lines

Over a period of several months a total of about 700 primary transformants harboring 3F-EAR T-DNA constructs were transferred to soil. After about 3 more weeks, trays holding up to 28 plants corresponding to the same 3F pool were photographed from the top and the RSA in pixel^2^ of every plant was calculated using the ‘Analyze Particles’ function of ImageJ. About 100 individuals that had ≥ 80% larger RSA than the tray average were allowed to complete their life cycle and their seeds (T2 progeny) were harvested.

For more detailed RSA quantification throughout development of selected lines, approximately 50 T2 seeds of each genotype and 200 for Col-0 were sown on soil and stratified for 3 days at 4°C. At 7 dpg, the largest individuals of each 3F-EAR line (n = 7) and Col-0 (n = 100) were transferred to soil in individual 67 x 67 x 65 mm pots (Pöppelmann, Lohne, Germany). In this manner, it was ensured that the growth of possibly larger 3F-EAR plants was always compared to the growth of the largest Col-0 individuals. From 10 dpg onwards and every 3 days, RSA was determined and the lines with ≥ 20% larger RSA than Col-0 were selected at 25 dpg.

### Construction of the collection of 3F-VP16 lines and screening

A library of approximately 3500 3F-VP16 fusion encoding T-DNA constructs was previously constructed in the binary vector pRF-VP16-Kana [19]. Five primary transformants (T1 plants) originating from the same subpool were placed together in a pot, and their seeds were together harvested and stored in seed bags named ‘five-bags’. Among a total of 1034 five-bags, a fraction contained less complicated seed mixtures (originating from 3 or 4 primary transformant rather than 5) due to losses of plants during cultivation and occasional infertility, the latter noticed in about 4% of the T1 plants. In total, the five—bags represented the T2 progeny of 4278 T1 plants. Approximately 20 seeds from each five-bag of the 3F-VP16 seed library were sown together on soil in a single pot. The seeds were stratified on soil for 3 days at 4°C, and every pot was inspected by eye at 14 dpg to identify plants with large rosettes compared to Col-0. These individuals were isolated, transferred to fresh soil and allowed to set seeds, which were harvested.

### Isolation of 3F encoding sequences from selected lines and retransformation with reconstituted T-DNA constructs

The 3F-EAR or 3F-VP16 encoding DNA fragments were amplified by PCR from the genomic DNA of the selected 3F-ATF lines with increased RSA using a forward primer within the *RPS5a* promoter sequence (5′-GCCCAAACCCTAAATTTCTCATC-3′) and a reverse primer within the *NOS* terminator sequence of the T-DNA construct (5′-CAAGACCGGCAACAGGAT-3′). The PCR products were sequenced by Sanger sequencing (Macrogen Europe). Reconstituted pRF-EAR-Kana and pRF-VP16-Kana binary vectors were generated with the 3F fragments from the PCR products as described previously [19]. The binary vector p35S-VP16-Kana was obtained by introduction of the CaMV *35S* promoter sequence as a *Xma*I-*Sac*I fragment into similarly digested pRF-VP16-Kana. Reconstituted p35S-VP16-Kana binary vectors were generated with PCR-derived 3F fragments. In the same fashion Col-0 plants were transformed with the reconstituted constructs as described previously [19]. Materials like specific vectors or specific plant lines can be acquired by sending a request to the corresponding author.

### RSA and biomass quantification of selected 3F-ATF lines with increased RSA and retransformant lines harboring reconstituted 3F-ATF constructs

Approximately 50 seeds of the selected 3F-ATF lines (T2 for 3F-EAR and T3 for 3F-VP16; segregating except for VP16-02-003), the retransformant lines reconstituted from these lines (T2; segregating) and Col-0 were sown on soil. As described above, the largest Col-0, 3F-ATF and retransformant individuals were selected at 7 dpg and were each transferred to fresh soil in separate pots that were placed on a tray holding 18 transgenic individuals and 6 wild-type individuals. As every individual plant in such a grid of 24 pots is growing in the vicinity of either two (~ 17%), three (50%) or four (~33%) neighboring individuals, Col-0 plants were positioned in such a manner that these conditions were correspondingly represented among the Col-0 population. If every row of pots were assigned a letter (A-D), and every column a number (1–6), Col-0 individuals were grown at positions A3, B1, B5, C3, D1 and D5, respectively. The relative RSA compared to Col-0 was calculated by dividing the RSA of every individual by the average RSA of Col-0 and multiplying by 100%. Relative growth rates were calculated as described by Tessmer *et al*. [29]. For the quantification of biomass, the shoots of each plant were harvested at 28 dpg and fresh weight was determined. Dry weights were determined after 2 days of incubation at 60°C. All relative surface area, fresh weight and dry weight data were statistically analyzed for significant differences with Col-0 using the heteroscedastic T-Test function of Microsoft Excel 2010 (assuming unequal variance between samples). A *p*-value of 0.05 was used as a threshold for significance.

### RNA extraction for RNA sequencing

Approximately 100 seeds of Col-0 (universal control), the selected 3F-ATF lines with larger RSA than Col-0, and a mixture of lines originating from the same 3F pools as those lines, but which do not have noticeably increased RSA (referred to as ‘background control samples’) were sown on soil and stratified for 3 days at 4°C. The background control samples were included to allow for the identification of DEGs (differentially expressed genes) that are specific for a particular 3F-ATF expressing plant line rather than being triggered by a more generic 3F-ATF response. We chose to use three samples of different pools of 12 plants each for each plant line (therefore n = 3), in order to mitigate the effects of individual transgenic plants on the transcriptome data. We therefore followed the rationale that *in trans* acting 3F-ATFs are expected to exert dominant effects on the average transcriptomes of a collection of plants that segregate for transgene presence, while potentially interfering recessive traits due to T-DNA insertion knockout of genes should largely be masked. At 7 dpg, 36 swiftly developing individuals of each genotype were randomly picked and were transferred to individual pots in three replicates with 12 individuals per replicate. All pots were randomly distributed over trays in the greenhouse to mitigate the effect of local variation in growth conditions on the transcriptome data. The aboveground parts of each replicate of 12 plants were combined and ground to powder in liquid nitrogen with pestle and mortar at 15 dpg. Care was taken to collect the plant material in a random order between 4 and 5 hours after the onset of light. RNA was extracted from 50–100 mg of tissue powder of each replicate using the RNeasy Plant Mini Kit (Qiagen).

### RNA sequencing data analysis

A sequencing library was constructed and sequenced by Illumina sequencing (50 cycles; 50 bp single reads). Genomic reference sequences and annotations were obtained from TAIR (version TAIR10) and supplemented with fragments mapping to VP16 from pRF-VP16-Kana and EAR from pRF-EAR-Kana. We acquired raw transcriptome data that allowed for the calculation of fold-change values for 12546 genes. The splicing-aware aligner TopHat version 2.0.10, [30] was used to map reads, using the ‘very-sensitive’ and ‘coverage-search’ options, and allowing for a maximum intron size of 15000 bp. Secondary alignments were removed from the BAM files using SAMtools (version 0.1.18, [31]) and Perl. Reads aligning to annotated exons were summarized at the level of TAIR genes using HTSeq (version 0.5.3p9, [32]) using the ‘intersection-strict’ setting. At least 91% of raw sequencing reads could be uniquely assigned to a gene. Read counts were processed in R (version 3.0.2) using the edgeR package (version 3.4.2, [33]). Normalized expression values per gene (excluding mitochondrial and chloroplast sequences) were obtained by scaling using a robust estimate of the library size [34] and dividing by the mean length of the annotated transcripts in kbp. A list of all annotated genes and their normalized expression values was uploaded to NCBI as BioProject (BioProject ID: PRJNA290552). For unknown reasons one of the three replicates of VP16-05-014 had such a large sample-sample distance to the other two replicates that it was disregarded for the data analysis described in this paper. Active gene expression was distinguished from background noise using the method of Hart *et al*. [35]. For the determination of differentially expressed genes (DEGs) raw *p*-values were adjusted for multiple testing with the Benjamini-Hochberg false discovery rate (FDR) procedure. DEGs were determined using the Bioconductor R package DESeq2 [36]. When comparing the transcriptomes of the transgenic lines to those of Col-0, a relatively large number of genes could be marked as differentially expressed when using an adjusted *p*-value of 0.05 as a threshold for significance. When adding 2-fold up- or down-regulation as an additional criterion for significance, about 90% of the DEGs were lost, but at the cost of also removing relatively highly expressed genes that do not meet the 2-fold criterion, but whose changes are extremely significant and contribute to GO enrichment. As a compromise, genes were considered DEGs if their adjusted *p*-value was lower than 0.0001. The resulting list of DEGs was used as input for the PANTHER classification tool [37] with default parameters. GO enrichment analysis was performed using the online tool of the Gene Ontology Consortium [38]. All the GO terms from the biological process category with a Bonferroni corrected *p*-value lower than 0.05 were considered as significant.

### RT-qPCR analysis

Seedlings of Col-0, VP16-02-003 (T3 generation), and the retransformant lines harboring 3F-VP16 constructs reconstituted from VP16-02-003 under control of either the *RPS5a* promoter or the *CaMV* 35S promoter (T2 generation; segregating) were grown as described above. At 15 dpg Shoots of pools of 12 plants were combined and ground to powder in liquid nitrogen with pestle and mortar. RNA was extracted from 50–100 mg of tissue powder of each pool using the RNeasy Plant Mini Kit (Qiagen). First-strand cDNA synthesis was performed using the iScript Select cDNA Synthesis Kit (BIORAD). RT-qPCR reactions were prepared using the SYBR Green PCR Master Mix (Applied Biosystems). PCR reactions were performed using the CFX96 Touch Real-Time PCR Detection System (BIORAD) using primer combinations specific for either the reference genes *ATG6* (At3g61710) and *SCAMP5* (At1g32050), generating 101 bp amplicons as described by Hruz *et al*. [39], or a forward (5′-ATTTACCCCCCACGACTCC-3′) and reverse (5′-ACCACCGTACTCGTCAATTC-3′) primer combination for the 3F-VP16 encoding gene from VP16-02-003, generating an amplicon of 103 bp. The expression values of the 3F-VP16 encoding genes in each of the transgenic lines were normalized to the average of the expression values of both reference genes. The QPCR data can be found in the supplement (S1 Dataset).

## Results

### Construction of the collections of 3F-ATF expressing Arabidopsis lines

In order to obtain collections of plant lines expressing different ZF-ATFs, a previously generated library of 3F constructs was used [19]. In brief, this library was composed of 15 non-overlapping subpools, each of them maximally consisting of 256 3F constructs. Each of the subpools was named after one of the sixteen 5’-GNN-3’ binding ZFs that was first cloned. A 16^th^ pool (with the 5’-GAA-3’ binding ZF as founder) proved to be unstable for unknown reasons [19], and was therefore not constructed. The 3F encoding sequences were translationally fused to the VP16 transcriptional activation domain or the EAR domain by cloning them into the pRF-VP16-Kana binary vector [19] or a similar vector designated pRF-EAR-Kana, respectively.

The collection of Arabidopsis lines expressing 3F-VP16 fusions was generated by transformation of Col-0 plants with each of the 15 subpools separately. Over an extended period of time, a total of approximately 5400 viable primary transformants were obtained, more or less evenly distributed over the 15 different 3F-VP16 subpools. As it has been shown that 6–30% of transformants can contain multiple T-DNAs originating from different bacteria after floral dip transformation [40], each primary transformant (T1 generation) can harbor any of the maximally 256 different 3F-ATF encoding genes present in each subpool, or more than one in the case of simultaneous transformation with another construct. Obtaining proof that a particular 3F-ATF causes a phenotype of interest therefore requires further experimentation by retransformation with single selected constructs, a procedure which is described in more detail below.

The large majority of the 3F-VP16 expressing T1 plants did not exhibit conspicuous growth phenotypes at standard growth conditions. The handling complexity of the collection was reduced by cultivating five primary transformants originating from the same subpool together in a single pot and collecting the T2 progeny of these plants in a total number of 1034 seed bags named ‘five-bags’. Assuming that T1 plants are heterozygous for a particular T-DNA insertion, it is important to note that their T2 offspring will exhibit Mendelian segregation for the 3F-VP16 encoding transgene; the T3 generation derived from individual transgenic T2 plants can be hemizygous or homozygous. Each line derived from a selected plant was assigned a code name consisting of three parts: effector domain– 3F pool number—five-bag of origin.

In order to assess the merits of 3F-EAR encoding gene constructs for genome interrogation, we decided to generate a relatively small collection of plant lines, representing just 7 different 3F-EAR subpools. A deliberately unsaturated screening of just about 100 primary transformant lines for each subpool should then give sufficient insight in the capacity of 3F-EAR proteins to induce phenotypic changes, while ensuring that most of the primary transformants express a different one of the 256 possible 3F-EAR fusions. Therefore, a total of just over 700 viable primary transformants were cultivated and their growth was documented as described below. Subsequently, the seeds (T2 generation) of individual T1 plants were harvested and were assigned a code name as mentioned above (T2 lines).

### Selection of 3F-ATF lines with increased rosette surface area

In this study, rosette surface area (RSA) was used as a measure for growth, because it is a non-destructive proxy for biomass in Arabidopsis [41]. For selection and further study of 3F-ATF induced phenotypes, it is important to note that the concept of genome interrogation depends upon the dominant and *in trans* activity of the 3F-ATFs. Furthermore, since more than one type of 3F-ATF encoding gene construct might be present in an original transformant, a phenotype of interest can only be attributed to the activity of a specific 3F-ATF encoding gene construct when transformation of wild-type plants with single selected gene constructs retrieved from the original transformants again produces plants or plant lines exhibiting this phenotype. Through their dominant nature, 3F-ATF induced phenotypes can already become visible in T1 plants. However, *in vitro* selection procedures for obtaining antibiotic or herbicide resistant T1 plants often distort plant development. For rather subtle and/ or quantitative traits such as RSA, initial observations should be followed by analysis of a population of unselected T2 plants, grown on soil or substrate directly; although ¼ of the population can be expected to be essentially wild-type, lacking a transgene, the dominant nature of 3F-ATF induced phenotypes should ensure that phenotypes of interest will be noticed when just assessing mean values of different populations. A further advantage of screening at the level of segregating populations will be that phenotypic effects of recessive insertion mutations will be mitigated, as only ¼ of the plants might exhibit such a recessive phenotype.

**Fig 1:**
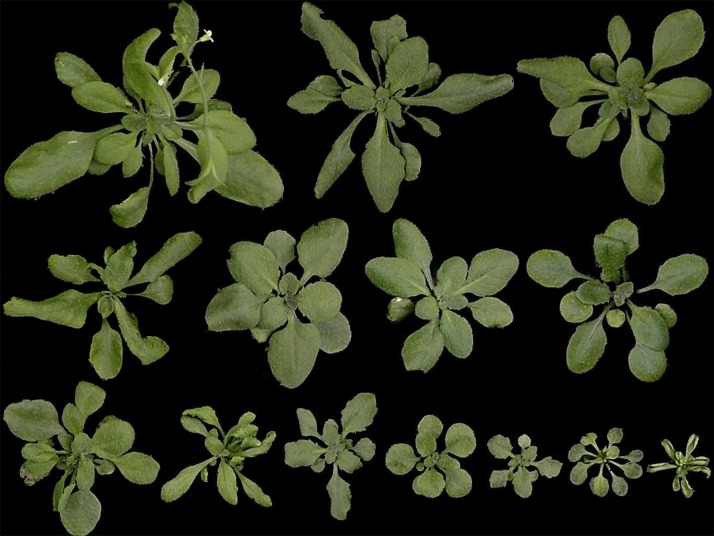
Variation in rosette phenotypes and growth characteristics among a population of primary transformants (T1) harboring 3F-EAR encoding T-DNA constructs. The presented individuals are representative of the extent of variation, but not in a quantitative manner. The plants were first grown on selection medium containing kanamycin, and were transferred to soil after approximately 2 weeks. The presented individuals are approximately 1 month old. The size of the individual in the right top corner is representative of a wild-type Col-0 plant at this stage of development. A truly wild-type Col-0 plant would not have survived the *in vitro* selection procedure used to obtain the transgenic plants, and was therefore not included in this Figure.

**Fig 2:**
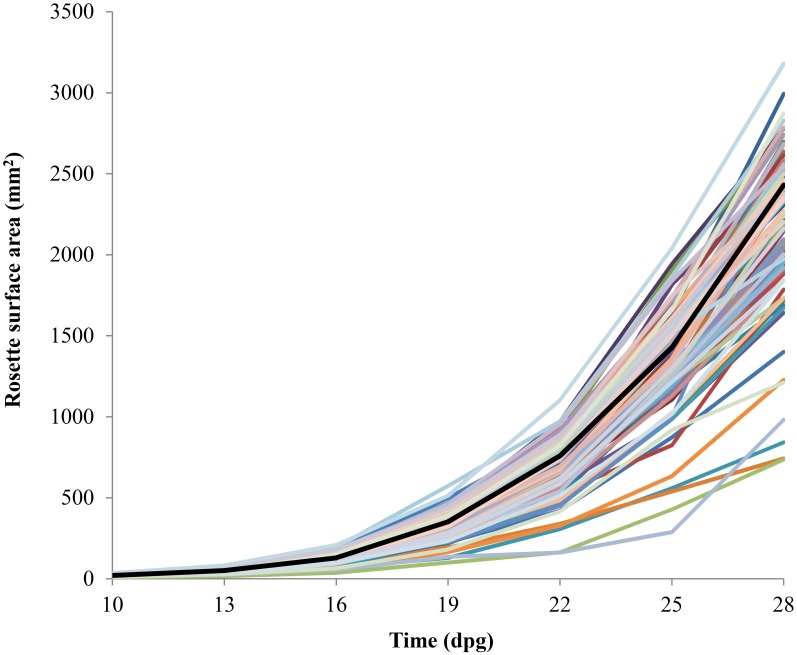
Growth curves of the T2 progeny (segregating) of the 100 largest 3F-EAR primary transformants in terms of RSA (n = 100 for Col-0; n = 7 for the transgenic lines). The growth curve of the Col-0 is presented in black.

Among the primary transformants harboring 3F-EAR encoding constructs there was great variation in rosette size, phenotype, leaf morphology and flowering time (Fig 1), clearly indicating that 3F-EAR fusions can induce phenotypic variation in Arabidopsis. To quantitatively assess the growth of 3F-EAR plants, we firstly quantified the RSA of all 700 primary transformants. Because the T1 plants were obtained through kanamycin selection, their growth could not be compared to similarly grown Col-0 plants, as these cannot be selected for with kanamycin. Also, not all plants could be raised and assessed simultaneously. Therefore, we decided to grow and compare plants to each other in batches of 28 individuals. In this way, approximately 100 primary transformants with at least 80% larger RSA than the batch average were selected for further analysis. Subsequently, the RSA of a segregating population of their T2 progeny was quantified throughout development. Again, a substantial amount of variation was observed in the RSA of the T2 progeny (Fig 2). For approximately one third of the 100 selected lines the mean RSA values were also larger than Col-0 in the T2 generation. Four lines that displayed the most substantial increases in RSA were selected for further analysis as described below.

Overall, there was much less phenotypic variation among 3F-VP16 plants compared to 3F-EAR plants. Approximately 5% of the 3F-VP16 primary transformants exhibited clearly visible phenotypic differences with Col-0 in terms of for instance leaf morphology, leaf color and flowering time. When further investigated in the T2 generation, such conspicuous phenotypes often segregated in a 3:1 ratio, indicating that the phenotypes were indeed inherited in a dominant manner as expected when induced by ZF-ATFs. Since we did not collect RSA data for the seven-fold larger collection of 3F-VP16 T1 plants and for practical reasons did not store offspring of each T1 plant separately, we decided to screen for 3F-VP16 plants with relatively large RSA in a pragmatic fashion in the T2 generation, just aiming for plants with the most extreme increases in RSA. Such increases were noted in the lines VP16-02-003 and VP16-05-014 and we therefore focused on further analyzing the T3 offspring of these lines, as described below.

### Quantification of RSA and further analysis of selected 3F-ATF lines

**Fig 3:**
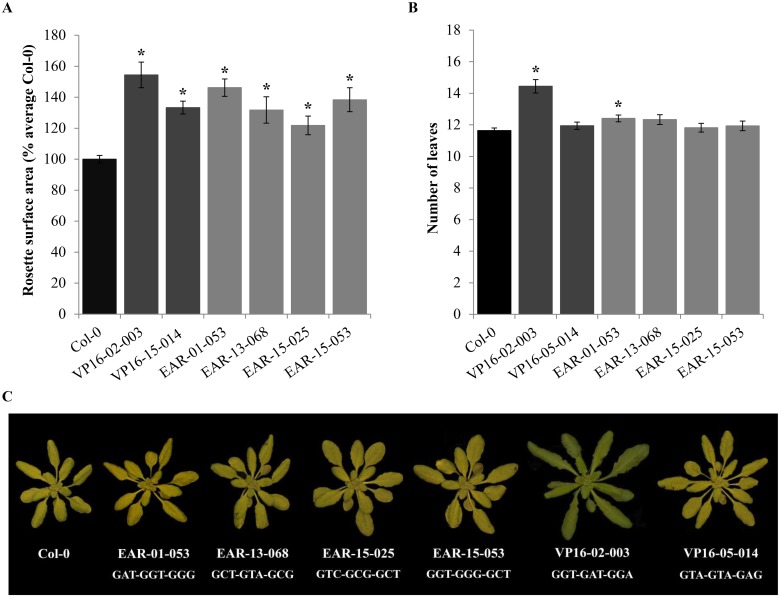
**A**) Quantification of the relative RSA of the selected 3F-EAR lines (T2; segregating) and 3F-VP16 lines (T3; only VP16-05-014 segregating) compared to Col-0 at 25 dpg. The RSA of each plant was calculated in terms of percentage of the average of Col-0. Error bars represent SEM values (n = 182 for Col-0, n = 16–18 for the transgenic lines). Significant differences with the Col-0 are indicated by an * (*p* < 0.05). **B**) Number of true leaves with discernable petioles at 25 dpg. Error bars represent SEM values (n = 36 for Col-0, n = 16–18 for the transgenic lines). Significant increases compared to Col-0 indicated by asterisks (*) (*p* < 0.05). **C)** Overview of the rosette phenotypes of the selected 3F-ATF lines (25 dpg), and of the 9 bp DNA recognition sequences of the 3Fs that were isolated from them. The presented individuals had RSA values closest to the average value found for their genotypes.

Altogether four 3F-EAR lines (T2 generation) and two 3F-VP16 lines (T3 generation) with substantially larger RSA than Col-0 were selected from the 3F-ATF libraries. Subsequently, a comparative analysis of RSA was performed to verify this selection and to assess the performance of each of these 3F-ATF lines compared to Col-0 in more detail. The overall increase in RSA of the lines compared to Col-0 ranged from approximately 20% to 55% at 25 dpg (Fig 3A). In terms of leaf number at 25 dpg, VP16-02-003 plants showed a significant and substantial increase of 24% compared to Col-0 plants (Fig 3B), indicating that the increase in RSA is for a great deal attributable to faster rosette development. For the other lines, however, the increase in leaf number was less than 7%, indicating that the RSA increases were predominantly caused by increases in leaf area.

**Fig 4:**
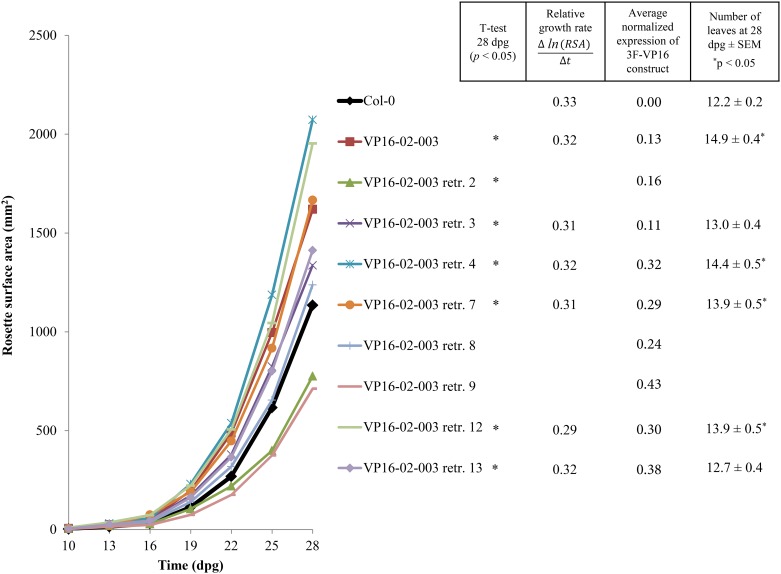
Growth curves of the wild-type Col-0, VP16-02-003 (T3) and retransformants reconstituted from VP16-02-003 (T2; segregating) (n = 48 for Col-0, n = 15–18 for the transgenic lines). Significant differences with Col-0 at 28 dpg are indicated by an * (*p* < 0.05). For each line the average normalized expression values of the 3F-VP16 construct as determined by RT-qPCR analysis at 15 dpg are provided. For the lines with significantly larger RSA than Col-0 the average relative growth rate between 10 and 28 dpg and the total number of with discernable petioles at 28 dpg are provided. Significant differences with Col-0 are indicated by an * (*p* < 0.05).

Sequence analysis of PCR products that were retrieved from the six selected larger plant lines showed that all lines contained 3F-ATF encoding genes with different predicted DNA recognition sites (Fig 3C). T3 populations of VP16-02-003 plants had a remarkably uniform appearance, with all individuals being substantially larger than Col-0 throughout development (Fig 4). We also did not observe segregation for kanamycin resistance among T3 VP16-02-003 plants, indicating that the original VP16-02-003 plant that was isolated in the T2 generation must have already been homozygous for the T-DNA insertion involved in enhanced RSA. VP16-02-003 plants started bolting at approximately 28 dpg at a 12 h photoperiod (one week earlier than Col-0) and developed substantially larger inflorescences which produced only few viable seeds. Although VP16-02-003 plants had a much higher yield in terms of RSA, this was therefore not reflected by seed yield. All other transgenic plant lines bolted later than 28 dpg at about the same time as Col-0 plants, and had normal seed set.

### The effect of the isolated 3F-ATF encoding T-DNA constructs on the growth of Arabidopsis

To investigate whether the 3F-ATF encoding genes from the six selected lines (Fig 3) were indeed the causative agents for increased RSA, reconstituted T-DNA constructs were generated harboring the 3F sequences that had been isolated from these lines. Col-0 plants were transformed with these reconstituted T-DNA constructs and RSA of a segregating population of T2 retransformant plants was analyzed throughout development. The T2 retransformant lines harboring reconstituted 3F-EAR constructs did not exhibit any clear increases in RSA compared to Col-0, indicating that they were not the direct causative agents for increased RSA, or that they induced RSA in the original mutant lines in a rather intricate manner possibly dependent on the genomic T-DNA integration locus.

Five out of the eight retransformant lines reconstituted from the original VP16-02-003 line displayed a larger RSA than Col-0 throughout development (Fig 4). At 28 dpg, these differences were in all cases significant at *p* < 0.05 (Fig 4), except for retransformant line 3, which had a larger RSA at *p* < 0.07. The significant increases in RSA were in the cases of three out of five retransformant lines reflected by significant increases in the total number of leaves at 28 dpg (Fig 4), indicating that the increase in RSA of these lines can be attributed to an increase in both leaf number and area, as was also found for the original VP16-02-003 line (Fig 3). The increases in absolute RSA were however not accompanied by significant changes in relative growth rate (Fig 4), indicating that the original VP16-02-003 line and the retransformant lines do not grow faster than Col-0, but achieve an increase in RSA through the amplification of a small difference in RSA with the same relative growth rate. With the majority of the retransformant lines having a larger RSA than Col-0, and with the increases being comparable to or even higher than the increase noted for the original VP16-02-003 line, we concluded that the expression of the 3F-VP16 encoding T-DNA construct from VP16-02-003 induced an increase in the RSA of Arabidopsis plants. Among the different retransformant lines the ones with the largest RSA also had the lowest relative expression of the 3F-VP16 construct (Fig 4).

**Fig 5:**
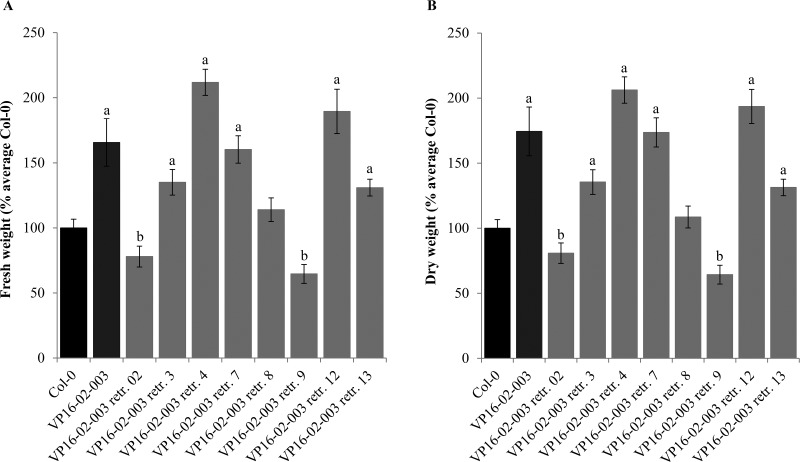
Quantification of the relative fresh weight (A) and the relative dry weight (B) of VP16-02-003 plants (T3, non-segregating) and retransformant plants reconstituted from VP16-02-003 (T2; segregating) compared to the Col-0 (28 dpg). The fresh and dry weights of each plant were calculated in terms of percentage of the average of Col-0. Error bars represent SEM values (n = 48 for Col-0, n = 15–18 for the transgenic lines). Significant increases compared to Col-0 are indicated by the letter ‘a’ (*p* < 0.05), and significant decreases are indicated by the letter ‘b’ (*p* < 0.05).

Among the T2 retransformant plants there was variation in leaf morphology, shape and number throughout development which started to become clearly visible between 13 and 16 dpg, and which can likely be attributed to segregation of the transgene. In contrast to the original VP16-02-003 line, all retransformant plants had wild-type levels of fertility. The retransformant lines that were significantly larger than Col-0 exhibited increases in RSA ranging from 20 to 80% when quantified at 28 dpg, and had an increased number of leaves which were slightly lanceolate in shape (S1A Fig). Among the population of T2 retransformant plants there were individuals that were substantially larger than the largest Col-0 individual in the population (S1B Fig), indicating that the 3F-VP16 construct from VP16-02-003 introduced variation in RSA reaching beyond the variation that can be produced by the Col-0 background genotype itself. Five out of the eight retransformant lines reconstituted from the original VP16-02-003 line also had a significantly higher relative fresh weight (Fig 5A) and dry weight (Fig 5B) than Col-0 at 28 dpg, thereby confirming the correlation between RSA and biomass. The average relative increases in fresh weight (Fig 5A) and dry weight (Fig 5B) of these T2 retransformant lines varied from approximately 30 to 110%, with the increases for the original VP16-02-003 line being 65% and 74%, respectively.

Through retransformation with a 3F-VP16 construct reconstituted from the original VP16-05-014 line (T3 generation) we found that two out of the five T2 retransformant lines constructed in this way displayed a significantly larger absolute RSA than Col-0 from 16 dpg and onwards (S2 Fig), which was also not accompanied by noticeable changes in relative growth rate. Therefore, these results indicated that the 3F-VP16 construct from VP16-05-014 is also capable of inducing an increase in RSA *in trans* and in a dominant manner. Analogous observations could be made in terms of fresh weight (S3A Fig) and dry weight (S3B Fig), in which cases the overall increases varied from approximately 4 to 30%, respectively. While almost all RSA increases were highly reproducible, it could sometimes not be demonstrated for VP16-05-014 segregating lines (S2 Fig) and was never found in a VP16-05-014 T3 line that did not segregate for T-DNA presence. Nevertheless, our data demonstrate that we have isolated two different and portable 3F-VP16 encoding T-DNA constructs that can induce a significant increase in both RSA and biomass in Arabidopsis.

### The effect of 3F-VP16 expression driven by the CaMV *35S* promoter

Both the VP16-02-003 and VP16-05-014 lines harbor 3F-VP16 encoding T-DNA constructs of which the expression is driven by the promoter of the Arabidopsis *RPS5a* gene, which is primarily active in meristematic tissue. As these constructs already induced substantial increases in both RSA and biomass, we considered it possible that constitutive overexpression of the 3F-VP16 fusions would lead to an even further enhancement of RSA and biomass. To investigate this, reconstituted 3F-VP16 encoding sequences from the original VP16-02-003 and VP16-05-014 lines were placed under control of the constitutive CaMV *35S* promoter, and the RSA of segregating populations of T2 retransformant plants harboring these constructs was quantified throughout development. Remarkably, the majority of these retransformant lines now had smaller RSA throughout development compared to Col-0 (S4 and S5 Figs), indicating that constitutive and/or high levels of expression of these 3F-VP16 fusions can lead to a negative rather than a positive effect on the growth of Arabidopsis. By means of RT-qPCR, we found that CaMV *35S* promoter driven expression led to 2- to 15-fold increases in 3F-VP16 transcript levels in T2 plants compared to when expression was driven by the *RPS5A* promoter (S4 Fig).

### Transcriptome analysis of 3F-EAR lines

**Fig 6:**
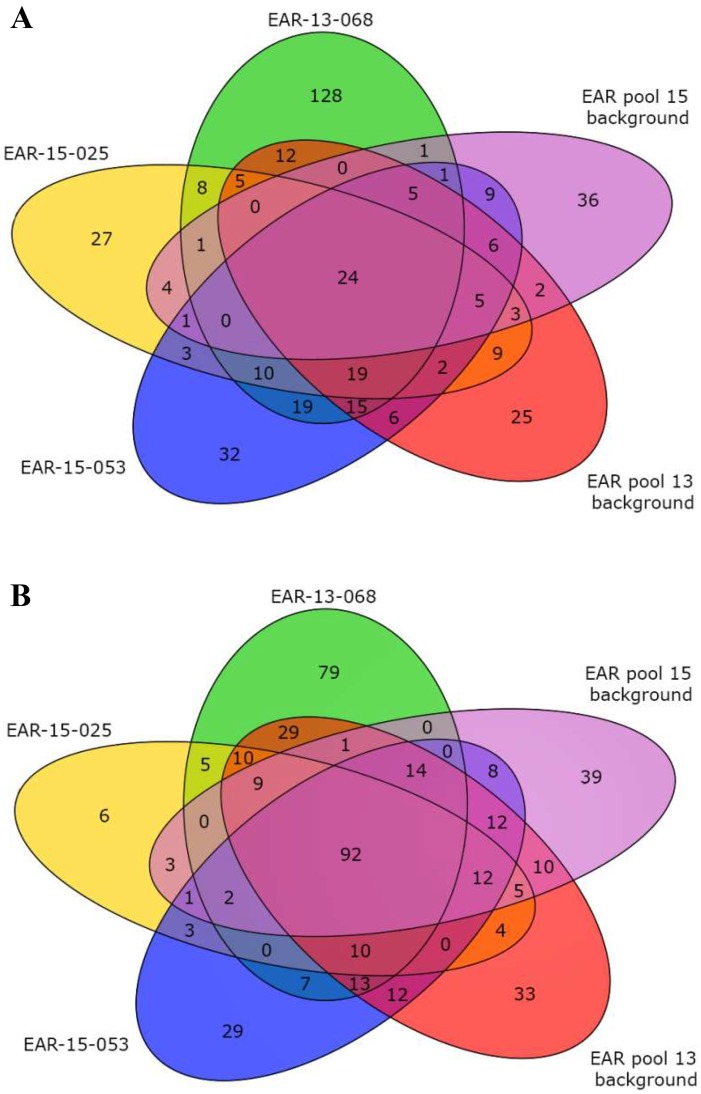
Venn diagrams of Differentially Expressed Genes (DEGs) compared to the Col-0 in the RNA sequencing data sets of the indicated 3F-EAR transgenic lines (*p* < 0.0001). ‘Background’ refers to RNA expression data derived from the pool of lines expressing 3F-EAR fusions similar to the specific 3F-EAR fusion expressed in the selected lines, but without a noticeable increase in RSA. **A**) Upregulated DEGs. **B**) Downregulated DEGs.

RNA sequencing was performed to investigate the 3F-ATF induced changes in gene expression patterns leading to the enhancement of RSA in the selected 3F-EAR lines. Line EAR-01-053 was randomly withdrawn from the transcriptome analysis, as it necessitated the sampling of a separate background pool. Of the three remaining lines, EAR-13-68, EAR-15-025 and EAR-15-053, the latter two could be compared to the same background pool. The transcriptomes of these three 3F-EAR expressing lines with increased RSA exhibited approximately 300 to 500 differentially expressed genes (DEGs) compared to Col-0. (Fig 6 and S1 Table). Similar numbers of DEGs compared to Col-0 were found for the background control samples of plants expressing a random selection of related 3F-EAR constructs (Fig 6 and S1 Table). When subtracting the cognate ‘background DEGs’ from the DEGs observed in the 3F-EAR lines with increased RSA, only about half of the DEGs remained (S1 Table). Hence, a large fraction of the transcriptomic changes in the 3F-EAR lines might have been triggered by expression of the EAR domain itself, regardless of the translational fusion to a 3F domain. In total 157 DEGs compared to Col-0 were shared between the transcriptomes of the three 3F-EAR lines with increased RSA, with the majority of them (116) also being shared by the two background samples (Fig 6). Remarkably, 33 of the 157 DEGs (21%) appeared to be nuclear encoded chloroplast proteins (S2 Table). Since 2039 of the 12546 expressed genes (16.5%), divided among all treatments in our transcriptomics data were annotated in the TAIR10 database as localized in the chloroplasts, apparently there has been some enrichment for the expression of chloroplast proteins. This enrichment becomes even more apparent if only the common DEGs of the original EAR and VP16 lines are taken into account (VP16-02-003, VP16-05-014, EAR-13-68, EAR-15-25 and EAR-15-53). This group consists of 30 downregulated genes and 4 upregulated genes (S2 Dataset). Of these 34 genes 28% are predicted to be present in chloroplasts.

Of the 157 DEGs that were shared between the 3F-EAR lines with increased RSA, 104 were upregulated and 53 were downregulated compared to Col-0 (S2 Table). GO enrichment analysis for the upregulated genes showed an almost exclusive enrichment for categories of genes directly or indirectly involved in photosynthesis and other light regulated processes (S3 Table). For the downregulated genes there was enrichment for just three categories involved in rhythmic or circadian processes (S3 Table). The enrichments of categories among the 116 DEGs shared by all 3F-EAR lines (S4 Table) was highly similar to that found for all 157 DEGs compared to Col-0 (S5 Table).

Only 10 of the 157 DEGs compared to Col-0 were specifically shared between the three 3F-EAR lines with larger RSA and were thus not found in the two background control pools (S2 Table). These 10 DEGs were all upregulated compared to Col-0, indicating that these genes are unlikely to be primary targets for 3F-EAR transcription factors, as these should mediate gene repression rather than activation. Seven of them (70%) were annotated as nuclear encoded chloroplast proteins while two other genes (At1g23310 and At3g147700) were likely to be involved in photorespiration and sucrose transport, respectively. Up to four of these genes (At4g32770, At4g34350, At5g52570, and At5g67030) might be involved in the broad sense of isoprenoid/terpenoid metabolism or more specifically in carotenoid/xanthophyll metabolism (S6 Table).

### Transcriptome analysis of 3F-VP16 lines

**Fig 7:**
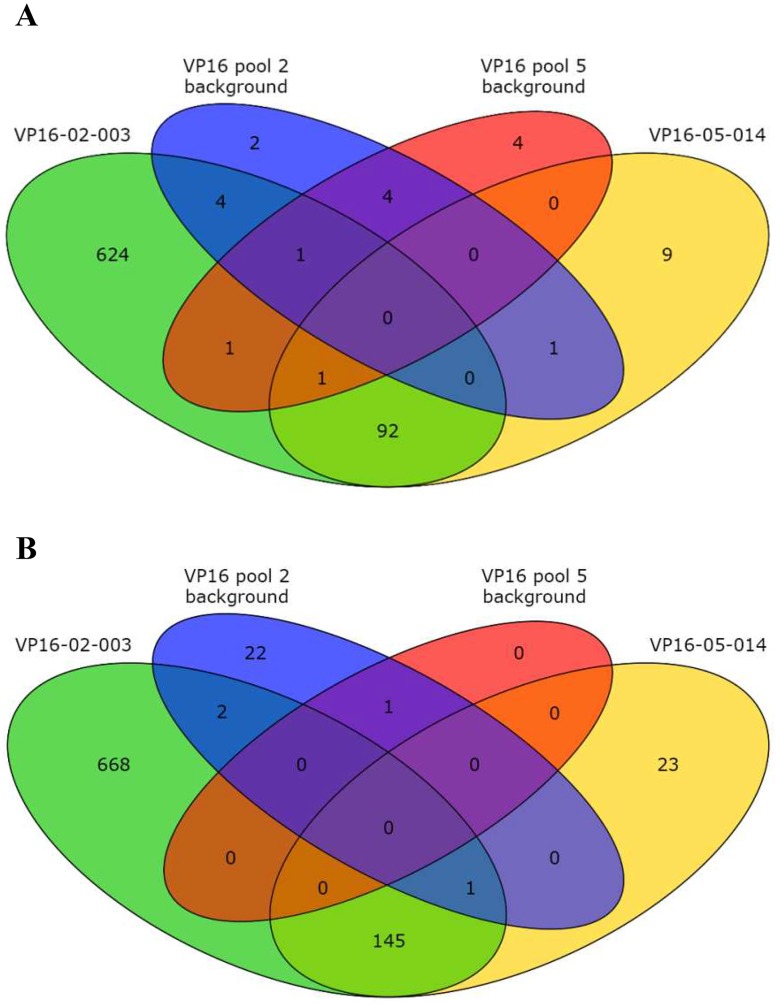
Venn diagrams of Differentially Expressed Genes (DEGs) compared to the wild-type Col-0 in the RNA sequencing data sets of the indicated 3F-EAR transgenic lines (*p* < 0.0001). ‘Background’ refers to RNA expression data derived from the pool of lines expressing 3F-VP16 fusions similar to the specific 3F-VP16 fusion expressed in the selected lines, but without a noticeable increase in RSA. **A**) Upregulated DEGs. **B**) Downregulated DEGs.

The 3F-VP16 lines VP16-02-003 and VP16-005-014 had 1539 and 272 DEGs compared to Col-0, respectively (Fig 7 and S7 Table). In contrast to the transcriptomes of the 3F-EAR lines, the number of background DEGs compared to Col-0 was very limited (Fig 7 and S7 Table). This clearly indicated that the transcriptomic changes in the 3F-VP16 lines were not so much due to the expression of the VP16 domain itself, but were predominantly caused by the activity of the complete 3F-ATF fusions. Subtraction of the cognate background DEGs made little difference in this respect, leaving 237 DEGs compared to Col-0 shared between VP16-02-003 and VP16-05-014 (Fig 7 and S7 Table). Several of the 3F-VP16 induced transcriptional changes that were found associated with an increase in RSA thus seem to be shared by VP16-02-003 and VP16-05-014, despite the fact that the predicted 9 bp DNA recognition sites of their 3F domains are very different (Fig 3C). As the presence of both different cognate 9 bp target sequences in the regulatory regions of the corresponding genes is very unlikely, such shared DEGs might much rather reflect a correlation with the shared phenotype, in this case plants with larger RSA. When looking specifically at the occurrence of potential 3F target sites within the promoter regions of the DEGs noted for the VP16-02-03 and VP-16-05-14 lines compared to Col-0, the predicted GGT-GAT-GGA recognition site for VP16-02-003 occurred in total 5 times among 1539 DEGs, while the GTA-GTA-GAG site for VP16-05-014 occurred 4 times among 272 DEGs. These frequencies were not different from random occurrence. When the DEGs shared by VP16-05-014 and VP16-02-003 were searched with the ZF recognition sites of VP16-05-014 and VP16-02-003 allowing for mismatches within the GNN-triplets for either one or both the Ns, we also did not find any significant enrichment for a particular 9 bp sequence in their promoters (S3 Dataset).

The 237 shared DEGs (S8 Table) were subjected to GO enrichment analysis. For the 146 DEGs that were upregulated compared to Col-0, the enriched GO categories were rather broadly defined (S9 Table), giving few definitive clues about the type of changes that are directly causal for an increase in RSA. GO analysis was much more outspoken for the 93 downregulated genes, where 4 out of 12 categories could be assigned to pathways involving sulfate reduction and synthesis of sulfur containing compounds (S9 Table). Obviously, these genes are unlikely primary target genes, as the initial 3F-VP16 induced transcriptional changes transcriptional changes are likely to be stimulatory.

## Discussion

In this study, we have described the construction and phenotypic characterization of two collections of Arabidopsis genome interrogation lines harboring 3F-EAR and 3F-VP16 artificial transcription factor encoding genes. There was extensive variation in the phenotype and RSA of 3F-EAR plants which was accompanied by a multitude of transcriptomic changes. In the case of two 3F-VP16 lines we could show that the 3F-VP16 constructs were causal for a significant increase in RSA and biomass, most likely through highly specific 3F-VP16 induced transcriptomic changes.

Selection of primary T1 transgenic seedlings on antibiotic containing medium and subsequent transfer to soil can considerably interfere with normal plant development. Our screening strategy for the isolation of 3F-EAR mutant lines with increased RSA therefore relied on making an early selection of 100 out of 700 T1 plants with a markedly larger RSA (at least 80%) compared to the mean RSA value determined for a total batch of 28 simultaneously raised T1 plants harboring similar 3F-EAR expression constructs. This early selection was then followed by a second selection based on a comparison of the RSA of their T2 progeny with Col-0 plants when all plant lines were grown on soil without antibiotic selection. If RSA data obtained for T1 plants are indicative for RSAs observed for the T2 progeny, an enrichment for larger plant lines can be expected. Although we did not pursue further experimentation to investigate whether such an enrichment for larger plant lines after the first screening had indeed occurred, we were readily able to select four lines with substantial and significant RSA increases compared to Col-0 using this stepwise protocol. Despite the successful isolation of these lines, the majority of the T2 plants derived from the 100 initially selected T1 plants did not exhibit larger RSA compared to Col-0. This might be best explained by the fact that the expression of 3F-EAR proteins has a generic negative effect on plant growth. In that manner, 3F-EAR expression constructs which promote growth have to overcome these negative effects before they can stimulate plant growth compared to Col-0. Our transcriptome data also suggest that expression of EAR fusion proteins can lead to shared gene expression changes, as further discussed below. The possibility of a generic negative effect of the EAR domain on plant growth can be of interest to researchers that apply CRES-T (Chimeric REpressor gene Silencing Technology [42]) to investigate the role of transcription factors by expressing translational fusions with an EAR (SRDX) domain, turning these transcription factors into negative regulators of cognate target genes. Most likely due to experimental differences regarding the source of tissues and the promoters used for transgene expression, meta-analysis of our transcriptomics data and those published by others for plants expressing EAR fusion proteins did not reveal any shared DEGs.

The screening of the 3F-VP16 library for lines with RSA phenotypes was more pragmatic, just aimed at the isolation of the largest plants by visual and by no means allowing for any quantitative assessment of the frequency of larger RSA phenotypes. While performing this screening, observed relatively little phenotypic and RSA variation among the plants of this library in comparison to the 3F-EAR library. Considering its history (allowing T1 plants to set T2 seed before doing any screening) it is possible that the T2 population of 3F-VP16 plants was enriched for 3F-ATF constructs with rather mild phenotypic effects because severely affected T1 plants did not or only marginally contribute to the T2 collection. Nevertheless, two 3F-VP16 lines with larger RSA than Col-0 were found in the present study and many several salt resistant lines in another screening [21], demonstrating that the collection of 3F-VP16 lines harbors very interesting mutant phenotypes.

The majority of the retransformant lines harboring T-DNA constructs that were reconstituted from the selected 3F-VP16 lines displayed an increase in RSA compared to Col-0, whereas the increases found for the selected 3F-EAR lines could not be reproduced by means of retransformation experiments. As already indicated above, growth promoting 3F-EAR constructs might have to overcome a generic negative effect of the EAR domain on plant growth before any increases over Col-0 plants can be noticed. In this respect, it is interesting to note that all investigated 3F-EAR lines with increased RSA and all background pools shared 116 DEGs compared to Col-0, which is about 30–40% of all DEGs observed (Fig 6 and S1 Table). These 116 DEGs were enriched for GO categories involved in photosynthetic and light regulated processes as well as circadian rhythm (S5 Table). One hypothesis could be that these DEGs are detrimental to plant growth. Alternatively, one could hypothesize that these generic changes in gene expression might help to set the stage for the development of larger plants when growth promoting 3F-EAR fusions are expressed. In both scenarios, the 10 DEGs compared to Col-0 that were specifically shared between the three selected plant lines with increased RSA (S2 and S6 Tables) might be examples of genes whose differential expression can contribute to the actual development of the phenotype. With 4 of the 10 DEGs involved in the pathway leading to xanthophyll biosynthesis (S6 Table), it is tempting to speculate that the presence of a particular xanthophyll or precursors thereof contributes to the development of larger plants. Xanthophylls are known to be involved in protecting the photosynthetic machinery from oxidative damage, most notably by quenching excess light energy, a process referred to as non-photochemical quenching [43]. In that manner, it could be envisaged that plant growth might be stimulated in the selected 3F-EAR expressing lines, but we have not yet further substantiated this lead. In the case of the selected 3F-VP16 lines, we were able to clearly provide the proof of principle of genome interrogation through retransformation with the reconstituted 3F-VP16 constructs. Although based on these observations it might be tempting to state that 3F-VP16 fusions are more useful for genome interrogation than 3F-EAR fusions, this would depend on the types of mutants screened for, the extent of phenotypic variation required to be generated and the experimental conditions used to perform the screening.

With the majority of the VP16-02-003 retransformant lines having a larger RSA than Col-0 proof was obtained that expression of the 3F-VP16 encoding T-DNA construct from VP16-02-003 can induce an increase in the RSA of Arabidopsis plants in a dominant manner (Fig 4). Since relative growth rate between 10 and 28 dpg were very similar, larger RSA values of the retransformant lines must therefore be attributed to enhanced growth during early developmental stages. Interestingly, the retransformant lines with the largest RSA also had the lowest relative expression of the 3F-VP16 construct at 15 dpg (Fig 4). The idea that higher expression levels of the construct might be counterproductive in terms of the induction of changes in growth was further corroborated using the CaMV *35S* promoter, where the highest expression levels were found in lines with the smallest RSA values (S4 Fig). Hence, it seems that the growth promoting effect of the 3F-VP16 construct is specifically exerted during an early stage of embryo or seedling development, a period in which the *RPS5A* promoter is very active. At later stages, *pRPS5A* driven gene expression is downregulated [23], thus prohibiting negative effects of untimely overexpression. Promoter choice can thus very much affect the outcome of genome interrogation experiments.

In the 3F-VP16 lines with increased RSA, we found a multitude of highly 3F specific transcriptomic changes while there were relatively few changes in the background control samples. Interestingly, the large majority (87%) of the original 272 DEGs noted for VP16-05-014 were shared by VP16-02-003 as DEGs that correlated with the development of larger plants.

While the shared upregulated genes did not provide clear leads towards further understanding the development of the larger phenotype, the downregulated gene genes were significantly enriched for genes involved in sulfate metabolism and the further synthesis of the sulfur containing glucosinolates (S8 Table). Most notably, the downregulated genes At4G14680 (*APS3*; ATP-sulfurylase 3), At2G14750 (*APK1*; adenyl sulfate kinase 1), and At4G39940 (*APK2*; adenyl sulfate kinase 2) are involved in the first steps of sulfate reduction and primary sulfate accumulation, processes essential for higher plant survival. However, these genes are members of small gene families with functional redundancy [44], and their downregulation in the two lines with increased RSA is far from complete, so severe phenotypes might not be expected. Regardless, it might be interesting to note that VP16-02-003 exhibited reduced seed set, a phenomenon that has been connected to sulfur deficiency [45]. However, as we did not observe reduced seed set in any of the retransformant lines, it might be that this particular aspect of the phenotype of the original line was caused by an extra mutation, such as an integrated T-DNA copy elsewhere in the genome. At present, we can only speculate why differential regulation of sulfur assimilation and glucosinolate biosynthesis can produce larger plants. Since sulfate reduction primarily occurs in chloroplasts [44] and preferably in the light [46], it might be that the reducing capacity of these organelles can now be more allocated to carbon fixation.

It should be noted that by analyzing the transcriptomes of the 3F-ATF lines at a fixed time point could mean that DEGs are found due to differences in developmental stage rather than being causal for RSA increases. Nevertheless, these types of changes ultimately reflect the phenotypic differences induced by 3F-ATF action. Also, there were clear differences between the 3F-VP16 lines and 3F-EAR lines with larger RSA in terms of enrichments for GO categories, meaning that increases in RSA can be achieved through different mechanisms. This is also likely to be due to the opposing activities of the EAR and VP16 effector domains. The overlap in DEGs among the 3F-EAR lines, and similarly so for the larger 3F-VP16 lines, could indicate that either the initial 3F-ATF triggered changes in gene expression were already partly overlapping, or that such an overlap started to occur further downstream. With hundreds of predicted potential binding sites for individual 3F domains being present in the Arabidopsis genome, an overlap of some primary target genes with recognition sites for more than one 3F domain in the gene control region is easily envisaged. Certainly so when taking in account that DNA recognition by ZF modules is not fully specific [22] and that many sites similar to the predicted target site might thus be recognized as well. Further channeling of downstream events by endogenous transcriptional regulators triggered along the process could then lead to a similar phenotype and a largely similar transcriptome.

One must expect that primary 3F-ATF target genes harbor a 3F binding site within their regulatory domains. Yet, analyses of 1000 bp upstream regions of DEGs for the occurrence of predicted 9 bp target sites or close derivatives thereof (S3 Dataset) did not show any significant enrichment for such sites, neither for the VP16-02-003 and VP16-05-14 lines, nor for the selected 3F-EAR lines where none of the 9 bp motifs occurred in the upstream regions of 3F-EAR specific DEGs. We can only speculate about the reason. First of all, the complete gene control region will often exceed the most proximal 1000 bp upstream region. Furthermore, important (causal) primary target genes might not have reached the threshold values for calling them expressed or differentially expressed. It should also be realized that target sites identified by *in silico* analysis can be unavailable for transcription factor binding *in vivo* due to a condensed chromatin structure.

The lack of enrichment for possibly expected GO categories that are immediately attributable to growth, such as cell expansion or cell division, might be surprising. However, finding GO categories via DEGs necessarily implies that a significantly large fraction of the entries for a category are also prone to differential gene expression, which might simply not be the case; genes belonging to a category can be rather constitutively expressed, not easily or never becoming DEGs. In that manner, our results illustrate that particular phenotypical characteristics, including transcriptomics data, are not necessarily in line with previously conceived ideas. Of course, genome interrogation is not the only strategy to discover plants with enhanced growth. Larger plants have also been discovered in EMS treated populations [47] or as consequence of heterosis [48]. In terms of efficacy, it is difficult to compare different methods, as the level of saturation of screenings might differ widely. Our study showed that larger plants can be obtained using genome interrogation, even by a rather pragmatic screening of a rather limited number of transformed plant lines. Moreover, 3F-VP16 encoding gene constructs proved to be portable elements that can simply confer the observed phenotype of interest to native plant lines in a dominant manner. Apart from generating the means to bring a particular phenotype under experimental control, genome interrogation can thus provide new avenues for further exploring plant development.

## Conclusions

Overall, we have isolated Arabidopsis lines with enhanced growth characteristics from both 3F-ATF libraries at relatively high frequencies. The use of genome interrogation enabled us to introduce variation in the growth and transcriptome of Arabidopsis and can in principle be applied to break through the figurative yield barrier of any plant species of interest.

## Supporting information

S1 Fig A to scale overview of the phenotypes and sizes of wild-type Col-0 plants, VP16-02-003 plants (T3) and plants of retransformant lines reconstituted from VP16-02-003 that have significantly larger RSA than the wild-type Col-0 (T2; segregating). **A**) Representative individuals of Col-0 (out of 48 plants), VP16-02-003 and retransformant lines harboring a T-DNA construct reconstituted from VP16-02-003. The transgenics that are larger than Col-0 have slightly lanceolate shaped leaves and exhibit an increase in the number of leaves (28 dpg). **B**) The largest individuals of the indicated genotypes in terms of RSA among the analyzed population of plants at 28 dpg.
(PDF)

S2 Fig Growth curves of the Col-0, VP16-05-014 (T3; segregating) and retransformants reconstituted from VP16-05-014 (T2; segregating) (n = 36 for Col-0, n = 18 for the other genotypes). In this experiment we were not able to reproduce the increase in RSA of VP16-05-014. Significant differences with Col-0 at 28 dpg are indicated by an * (*p* < 0.05). For each genotype the average relative growth rate is provided.
(PDF)

S3 Fig Quantification of the relative fresh weight (A) and dry weight (B) of wild-type Col-0 plants, VP16-05-014 plants (T3; segregating) and retransformant plants reconstituted from VP16-05-014 (T2; segregating) compared to the wild-type Col-0 (28 dpg). The fresh and dry weights of plants of the indicated genotypes was calculated in terms of percentage of the average of Col-0. Error bars represent SEM values (n = 36 for Col-0, n = 18 for the other genotypes). Significant differences with Col-0 are indicated by an * (*p* < 0.05). In this experiment we were not able to reproduce the increase in biomass of VP16-05-014.
(PDF)

S4 Fig Growth curves of the wild-type Col-0, VP16-02-003 (T3) and retransformants reconstituted from VP16-02-003 in the binary vector construct p35S-VP16-Kana (T2; segregating) (n = 84 for Col-0, n = 11–18 for the transgenic lines). Significant differences with Col-0 at 27 dpg are indicated by an * (*p* < 0.05). For each genotype the average relative growth rate is provided. For a selection of the genotypes the average normalized expression values of the 3F-VP16 construct as determined by RT-qPCR analysis is provided.
(PDF)

S5 Fig Growth curves of the wild-type Col-0, VP16-05-014 (T3) and retransformants reconstituted from VP16-05-014 in the binary vector construct p35S-VP16-Kana (T2; segregating) (n = 84 for Col-0, n = 11–18 for the transgenic lines). Significant differences with Col-0 at 27 dpg are indicated by an * (*p* < 0.05). For each genotype the average relative growth rate is provided.
(PDF)

S1 Table Overview of Differentially Expressed Genes (DEGs) compared to the wild-type Col-0 in the RNA sequencing data sets of the indicated 3F-EAR transgenic lines (*p* < 0.0001). ‘Background’ refers to RNA expression data derived from the pool of lines expressing 3F-EAR fusions similar to the specific 3F-EAR fusion expressed in the selected lines, but without a noticeable increase in RSA. ‘Overlap’ refers to the DEGs shared in a column.
(PDF)

S2 Table Overview of the 157 Differentially Expressed Genes (DEGs) compared to the wild-type Col-0 that are shared in the RNA sequencing data sets of the 3F-EAR transgenic lines EAR-13-68, EAR-15-025 and EAR-15-053 (*p* < 0.0001). The 10 DEGs that were not found in the transcriptomes of background pools are shaded in grey.
(PDF)

S3 Table Overview of significantly enriched GO categories (*p* < 0.05) found for the 104 upregulated (Up) and 53 downregulated (Down) DEGs compared to the wild-type Col-0 that are shared in the RNA sequencing data sets of the three larger 3F-EAR transgenic lines, EAR-13-68, EAR-15-025 and EAR-15-053. (PDF)

S4 Table Overview of the 116 Differentially Expressed Genes (DEGs) compared to the Col-0 that are shared in all RNA sequencing data sets derived from 3F-EAR transgenic lines and background pools (*p* < 0.0001). (PDF)

S5 Table Overview of significantly enriched GO categories (*p* < 0.05) found for the 24 upregulated (Up) and 92 downregulated (Down) DEGs compared to the Col-0 that are shared in the RNA sequencing data sets derived from 3F-EAR transgenic lines and background pools. (PDF)

S6 Table Overview of significantly enriched GO categories (*p* < 0.05) found for the selection of 10 out of 157 DEGs compared to the Col-0 that are shared in the RNA sequencing data sets of the three larger 3F-EAR transgenic lines, but are not found in the transcriptomes of background pools. (PDF)

S7 Table Overview of Differentially Expressed Genes (DEGs) compared to the Col-0 in the RNA sequencing data sets of the indicated 3F-VP16 transgenic lines (*p* < 0.0001). ‘Background’ refers to RNA expression data derived from the pool of lines expressing 3F-VP16 fusions similar to the specific 3F-VP16 fusion expressed in the selected lines, but without a noticeable increase in RSA. ‘Overlap’ refers to the DEGs shared in a column.
(PDF)

S8 Table Overview of the 239 Differentially Expressed Genes (DEGs) compared to the Col-0 that are shared in the RNA sequencing data sets of the two larger 3F-VP16 transgenic lines, VP16-02-003 and VP16-005-014 (*p* < 0.0001). The 2 DEGs that were also found in the transcriptomes of background pools are shaded.
(PDF)

S9 Table Overview of significantly enriched GO categories (*p* < 0.05) found for the 146 upregulated (Up) and 93 downregulated (Down) DEGs compared to the wild-type Col-0 that are shared in the RNA sequencing data sets of the two larger 3F-VP16 transgenic lines, VP16-02-003 and VP16-005-014. (PDF)

S1 Dataset Cq values of QPCR on Col-0, VP16-02-003 and the retransformant lines harboring 3F-VP16 constructs reconstituted from VP16-02-003 under control of either the *RPS5a* promoter or the *CaMV* 35S promoter. (XLSX)

S2 Dataset DEGs shared by the original EAR and VP16 lines. (XLSX)

S3 Dataset ZF motif search of DEGs shared by VP16-02-003 and VP16-05-014 allowing for one or two mismatches at the 2th and 3th nucleotide of each GNN-triplet. (XLSX)

## References

[1] Zhu XG, Long SP, Ort DR. Improving photosynthetic efficiency for greater yield. Annu Rev Plant Biol. 2010;61:235–61. 10.1146/annurev-arplant-042809-112206 20192734

[2] Wiebe K. The State of Food and Agriculture 2008 Biofuels: Prospects, Risks and Opportunities. Food and Agriculture Organization of the United Nations, Rome, Italy 2008.

[3] Peng SB, Tang QY, Zou YB. Current Status and Challenges of Rice Production in China. Plant Prod Sci. 2009;12(1):3–8.

[4] Evans JR. Improving photosynthesis. Plant physiology. 2013;162(4):1780–93. 10.1104/pp.113.219006 23812345 PMC3729760

[5] Barber J. Photosynthetic energy conversion: natural and artificial. Chemical Society reviews. 2009;38(1):185–96. 10.1039/b802262n 19088973

[6] Gonzalez N, Beemster GT, Inze D. David and Goliath: what can the tiny weed Arabidopsis teach us to improve biomass production in crops? Current opinion in plant biology. 2009;12(2):157–64. 10.1016/j.pbi.2008.11.003 19119056

[7] Gonzalez N, Vanhaeren H, Inze D. Leaf size control: complex coordination of cell division and expansion. Trends in plant science. 2012;17(6):332–40. 10.1016/j.tplants.2012.02.003 22401845

[8] Choe S, Fujioka S, Noguchi T, Takatsuto S, Yoshida S, Feldmann KA. Overexpression of DWARF4 in the brassinosteroid biosynthetic pathway results in increased vegetative growth and seed yield in Arabidopsis. Plant Journal. 2001;26(6):573–82. 11489171 10.1046/j.1365-313x.2001.01055.x

[9] Horvath BM, Magyar Z, Zhang YX, Hamburger AW, Bako L, Visser RG, et al EBP1 regulates organ size through cell growth and proliferation in plants. Embo J. 2006;25(20):4909–20. 10.1038/sj.emboj.7601362 17024182 PMC1618091

[10] Century K, Reuber TL, Ratcliffe OJ. Regulating the regulators: The future prospects for transcription-factor-based agricultural biotechnology products. Plant physiology. 2008;147(1):20–9. 10.1104/pp.108.117887 18443103 PMC2330319

[11] Meyer RC, Torjek O, Becher M, Altmann T. Heterosis of biomass production in Arabidopsis. Establishment during early development. Plant physiology. 2004;134(4):1813–23. 10.1104/pp.103.033001 15064384 PMC419853

[12] Groszmann M, Gonzalez-Bayon R, Greaves IK, Wang L, Huen AK, Peacock WJ, et al Intraspecific Arabidopsis hybrids show different patterns of heterosis despite the close relatedness of the parental genomes. Plant physiology. 2014;166(1):265–80. 10.1104/pp.114.243998 25073707 PMC4149712

[13] Andorf S, Meyer RC, Selbig J, Altmann T, Repsilber D. Integration of a systems biological network analysis and QTL results for biomass heterosis in Arabidopsis thaliana. PloS one. 2012;7(11):e49951 10.1371/journal.pone.0049951 23166802 PMC3500345

[14] Barth S, Busimi AK, Friedrich Utz H, Melchinger AE. Heterosis for biomass yield and related traits in five hybrids of Arabidopsis thaliana L. Heynh. Heredity. 2003;91(1):36–42. 10.1038/sj.hdy.6800276 12815451

[15] van Tol N, van der Zaal BJ. Artificial transcription factor-mediated regulation of gene expression. Plant Sci. 2014;225:58–67. 10.1016/j.plantsci.2014.05.015 25017160

[16] Lee JY, Sung BH, Yu BJ, Lee JH, Lee SH, Kim MS, et al Phenotypic engineering by reprogramming gene transcription using novel artificial transcription factors in Escherichia coli. Nucleic Acids Res. 2008;36(16):e102 10.1093/nar/gkn449 18641039 PMC2532725

[17] Park KS, Jang YS, Lee H, Kim JS. Phenotypic alteration and target gene identification using combinatorial libraries of zinc finger proteins in prokaryotic cells. J Bacteriol. 2005;187(15):5496–9. 10.1128/JB.187.15.5496-5499.2005 16030245 PMC1196030

[18] Park KS, Lee DK, Lee H, Lee Y, Jang YS, Kim YH, et al Phenotypic alteration of eukaryotic cells using randomized libraries of artificial transcription factors. Nat Biotechnol. 2003;21(10):1208–14. 10.1038/nbt868 12960965

[19] Lindhout BI, Pinas JE, Hooykaas PJ, van der Zaal BJ. Employing libraries of zinc finger artificial transcription factors to screen for homologous recombination mutants in Arabidopsis. Plant J. 2006;48(3):475–83. 10.1111/j.1365-313X.2006.02877.x 17052325

[20] Jia Q, van Verk MC, Pinas JE, Lindhout BI, Hooykaas PJ, van der Zaal BJ. Zinc finger artificial transcription factor-based nearest inactive analogue/nearest active analogue strategy used for the identification of plant genes controlling homologous recombination. Plant Biotechnol J. 2013;11(9):1069–79. 10.1111/pbi.12101 23915119

[21] van Tol N, P J.; Schat H.; Hooykaas P.J.; van der Zaal B.J. Genome interrogation for novel salinity tolerant Arabidopsis mutants. Plant Cell Environ. 2016. Epub 2016 Jul 26. 10.1111/pce.12805 27457432

[22] Segal DJ, Dreier B, Beerli RR, Barbas CF 3rd. Toward controlling gene expression at will: selection and design of zinc finger domains recognizing each of the 5'-GNN-3' DNA target sequences. Proceedings of the National Academy of Sciences of the United States of America. 1999;96(6):2758–63. 10077584 10.1073/pnas.96.6.2758 PMC15842

[23] Weijers D, Franke-van Dijk M, Vencken RJ, Quint A, Hooykaas P, Offringa R. An Arabidopsis Minute-like phenotype caused by a semi-dominant mutation in a RIBOSOMAL PROTEIN S5 gene. Development. 2001;128(21):4289–99. 11684664 10.1242/dev.128.21.4289

[24] Sadowski I, Ma J, Triezenberg S, Ptashne M. GAL4-VP16 is an unusually potent transcriptional activator. Nature. 1988;335(6190):563–4. 10.1038/335563a0 3047590

[25] Ohta M, Matsui K, Hiratsu K, Shinshi H, Ohme-Takagi M. Repression domains of class II ERF transcriptional repressors share an essential motif for active repression. The Plant cell. 2001;13(8):1959–68. 11487705 10.1105/TPC.010127 PMC139139

[26] Hiratsu K, Ohta M, Matsui K, Ohme-Takagi M. The SUPERMAN protein is an active repressor whose carboxy-terminal repression domain is required for the development of normal flowers. FEBS letters. 2002;514(2–3):351–4. 11943180 10.1016/s0014-5793(02)02435-3

[27] Hiratsu K, Matsui K, Koyama T, Ohme-Takagi M. Dominant repression of target genes by chimeric repressors that include the EAR motif, a repression domain, in Arabidopsis. The Plant journal: for cell and molecular biology. 2003;34(5):733–9. 12787253 10.1046/j.1365-313x.2003.01759.x

[28] de Pater S, Neuteboom LW, Pinas JE, Hooykaas PJ, van der Zaal BJ. ZFN-induced mutagenesis and gene-targeting in Arabidopsis through Agrobacterium-mediated floral dip transformation. Plant biotechnology journal. 2009;7(8):821–35. 10.1111/j.1467-7652.2009.00446.x 19754840

[29] Tessmer OL, Jiao Y, Cruz JA, Kramer DM, Chen J. Functional approach to high-throughput plant growth analysis. BMC Syst Biol. 2013;7 Suppl 6:S17. 24565437 10.1186/1752-0509-7-S6-S17 PMC4029786

[30] Kim D, Pertea G, Trapnell C, Pimentel H, Kelley R, Salzberg SL. TopHat2: accurate alignment of transcriptomes in the presence of insertions, deletions and gene fusions. Genome Biol. 2013;14(4). 10.1186/gb-2013-14-4-r36 PMC4053844 23618408

[31] Li H, Handsaker B, Wysoker A, Fennell T, Ruan J, Homer N, et al The Sequence Alignment/Map format and SAMtools. Bioinformatics. 2009;25(16):2078–9. 10.1093/bioinformatics/btp352 19505943 PMC2723002

[32] Anders S, Pyl PT, Huber W. HTSeq-a Python framework to work with high-throughput sequencing data. Bioinformatics. 2015;31(2):166–9. 10.1093/bioinformatics/btu638 25260700 PMC4287950

[33] Robinson MD, McCarthy DJ, Smyth GK. edgeR: a Bioconductor package for differential expression analysis of digital gene expression data. Bioinformatics. 2010;26(1):139–40. 10.1093/bioinformatics/btp616 19910308 PMC2796818

[34] Anders S, Huber W. Differential expression analysis for sequence count data. Genome Biol. 2010;11(10). 10.1186/gb-2010-11-10-r106 PMC3218662 20979621

[35] Hart T, Komori HK, LaMere S, Podshivalova K, Salomon DR. Finding the active genes in deep RNA-seq gene expression studies. BMC genomics. 2013;14:778 10.1186/1471-2164-14-778 24215113 PMC3870982

[36] Love MI, Huber W, Anders S. Moderated estimation of fold change and dispersion for RNA-seq data with DESeq2. Genome biology. 2014;15(12):550 10.1186/s13059-014-0550-8 25516281 PMC4302049

[37] Mi HY, Poudel S, Muruganujan A, Casagrande JT, Thomas PD. PANTHER version 10: expanded protein families and functions, and analysis tools. Nucleic Acids Res. 2016;44(D1):D336–D42. 10.1093/nar/gkv1194 26578592 PMC4702852

[38] Ashburner M, Ball CA, Blake JA, Botstein D, Butler H, Cherry JM, et al Gene Ontology: tool for the unification of biology. Nat Genet. 2000;25(1):25–9. 10.1038/75556 10802651 PMC3037419

[39] Hruz T, Wyss M, Docquier M, Pfaffl MW, Masanetz S, Borghi L, et al RefGenes: identification of reliable and condition specific reference genes for RT-qPCR data normalization. Bmc Genomics. 2011;12. 10.1186/1471-2164-12-156 PMC3072958 21418615

[40] De Buck S, Podevin N, Nolf J, Jacobs A, Depicker A. The T-DNA integration pattern in Arabidopsis transformants is highly determined by the transformed target cell. Plant J. 2009;60(1):134–45. 10.1111/j.1365-313X.2009.03942.x 19508426

[41] Leister D, Varotto C, Pesaresi P, Niwergall A, Salamini F. Large-scale evaluation of plant growth in Arabidopsis thaliana by non-invasive image analysis. Plant Physiol Bioch. 1999;37(9):671–8.

[42] Mitsuda N, Matsui K, Ikeda M, Nakata M, Oshima Y, Nagatoshi Y, et al CRES-T, an effective gene silencing system utilizing chimeric repressors. Methods Mol Biol. 2011;754:87–105. 10.1007/978-1-61779-154-3_5 21720948

[43] Jahns P, Holzwarth AR. The role of the xanthophyll cycle and of lutein in photoprotection of photosystem II. Bba-Bioenergetics. 2012;1817(1):182–93. 10.1016/j.bbabio.2011.04.012 21565154

[44] Kopriva S, Mugford SG, Baraniecka P, Lee BR, Matthewman CA, Koprivova A. Control of sulfur partitioning between primary and secondary metabolism in Arabidopsis. Front Plant Sci. 2012;3. 10.3389/fpls.2012.00163 PMC3400089 22833750

[45] Sorin E, Etienne P, Maillard A, Zamarreno AM, Garcia-Mina JM, Arkoun M, et al Effect of sulphur deprivation on osmotic potential components and nitrogen metabolism in oilseed rape leaves: identification of a new early indicator. J Exp Bot. 2015;66(20):6175–89. 10.1093/jxb/erv321 26139826

[46] Huseby S, Koprivova A, Lee BR, Saha S, Mithen R, Wold AB, et al Diurnal and light regulation of sulphur assimilation and glucosinolate biosynthesis in Arabidopsis. J Exp Bot. 2013;64(4):1039–48. 10.1093/jxb/ers378 23314821 PMC3580815

[47] Yue S, Zhang W, Li FL, Guo YL, Liu TL, Huang H. Identification and genetic mapping of four novel genes that regulate leaf development in Arabidopsis. Cell Res. 2000;10(4):325–35. 10.1038/sj.cr.7290059 11271002

[48] Kusterer B, Muminovic J, Utz HF, Piepho HP, Barth S, Heckenberger M, et al Analysis of a triple testcross design with recombinant inbred lines reveals a significant role of epistasis in heterosis for biomass-related traits in Arabidopsis. Genetics. 2007;175(4):2009–17. 10.1534/genetics.106.069005 17287529 PMC1855122

