# Supplementary material for: Enhancement of Arabidopsis growth characteristics using genome interrogation with artificial transcription factors
Source: PLoS One. 2017 Mar 30;12(3):e0174236. doi: 10.1371/journal.pone.0174236 (PMC5373528; doi:10.1371/journal.pone.0174236)
Supplement: S1 Fig — A) Representative individuals of Col-0 (out of 48 plants), VP16-02-003 and retransformant lines harboring a T-DNA construct reconstituted from VP16-02-003. The transgenics that are larger than Col-0 have slightly lanceolate shaped leaves and exhibit an increase in the number of leaves (28 dpg). B) The largest individuals of the indicated genotypes in terms of RSA among the analyzed population of plants at 28 dpg. (PDF) [file pone.0174236.s001.pdf]

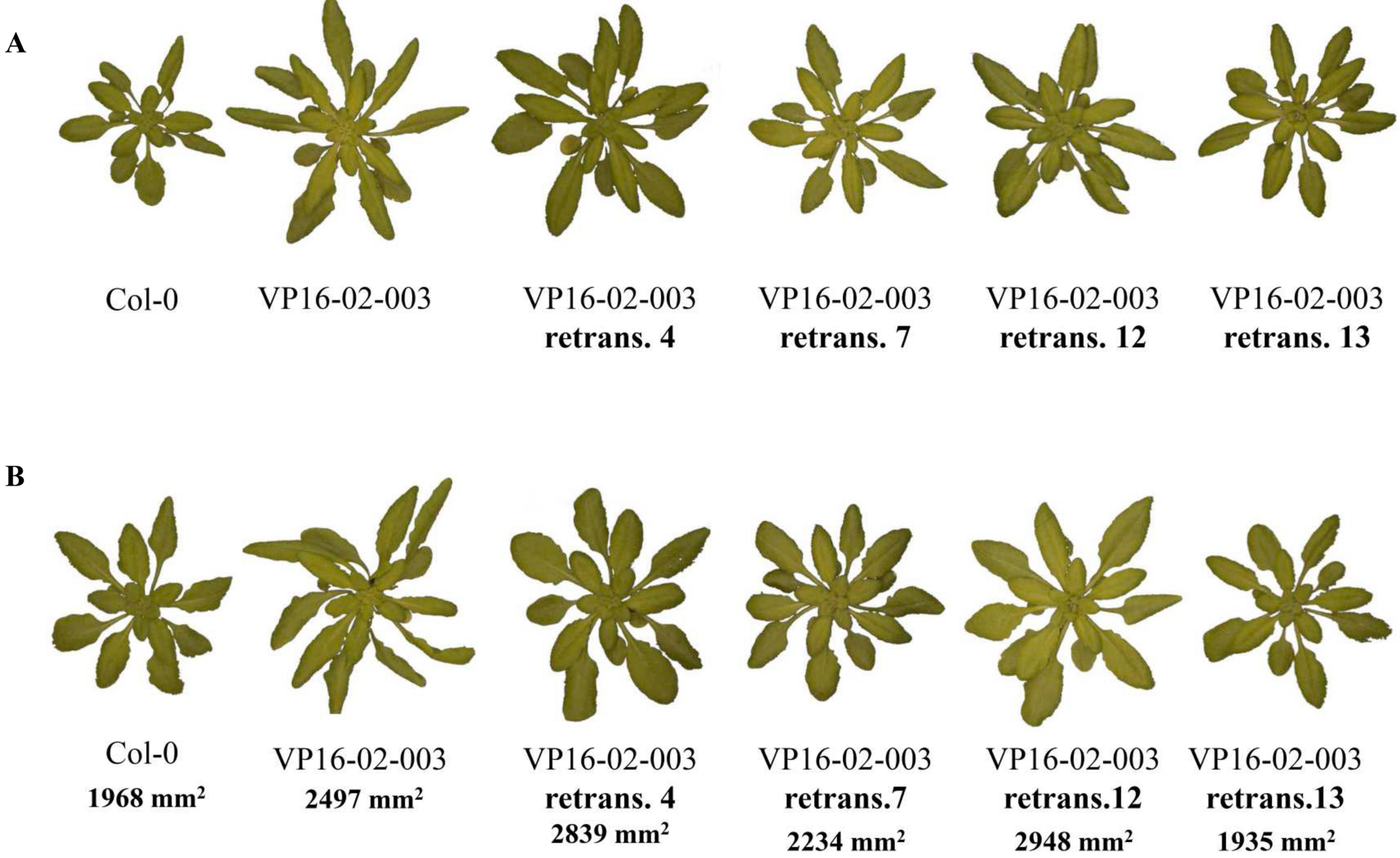

S1 Fig. A to scale overview of the phenotypes and sizes of wild type Col-0 plants, VP16-02-003 plants (T3) and plants of retransformant lines reconstituted fromVP16-02-003 that have significantly larger RSA than the wild type Col-0 (T2; segregating). **A**) Representative individuals of Col-0 (out of 48 plants), VP16-02-003 and retransformant lines harboring a T-DNA construct reconstituted from VP16-02-003. The transgenics that are larger than Col-0 have slightly lanceolate shaped leaves and exhibit an increase in the number of leaves (28 dpg). **B**) The largest individuals of the indicated genotypes in terms of RSA among the analyzed population of plants at 28 dpg.
