# Supplementary material for: Enhancement of Arabidopsis growth characteristics using genome interrogation with artificial transcription factors
Source: PLoS One. 2017 Mar 30;12(3):e0174236. doi: 10.1371/journal.pone.0174236 (PMC5373528; doi:10.1371/journal.pone.0174236)
Supplement: S2 Fig — In this experiment we were not able to reproduce the increase in RSA of VP16-05-014. Significant differences with Col-0 at 28 dpg are indicated by an * (p < 0.05). For each genotype the average relative growth rate is provided. (PDF) [file pone.0174236.s002.pdf]

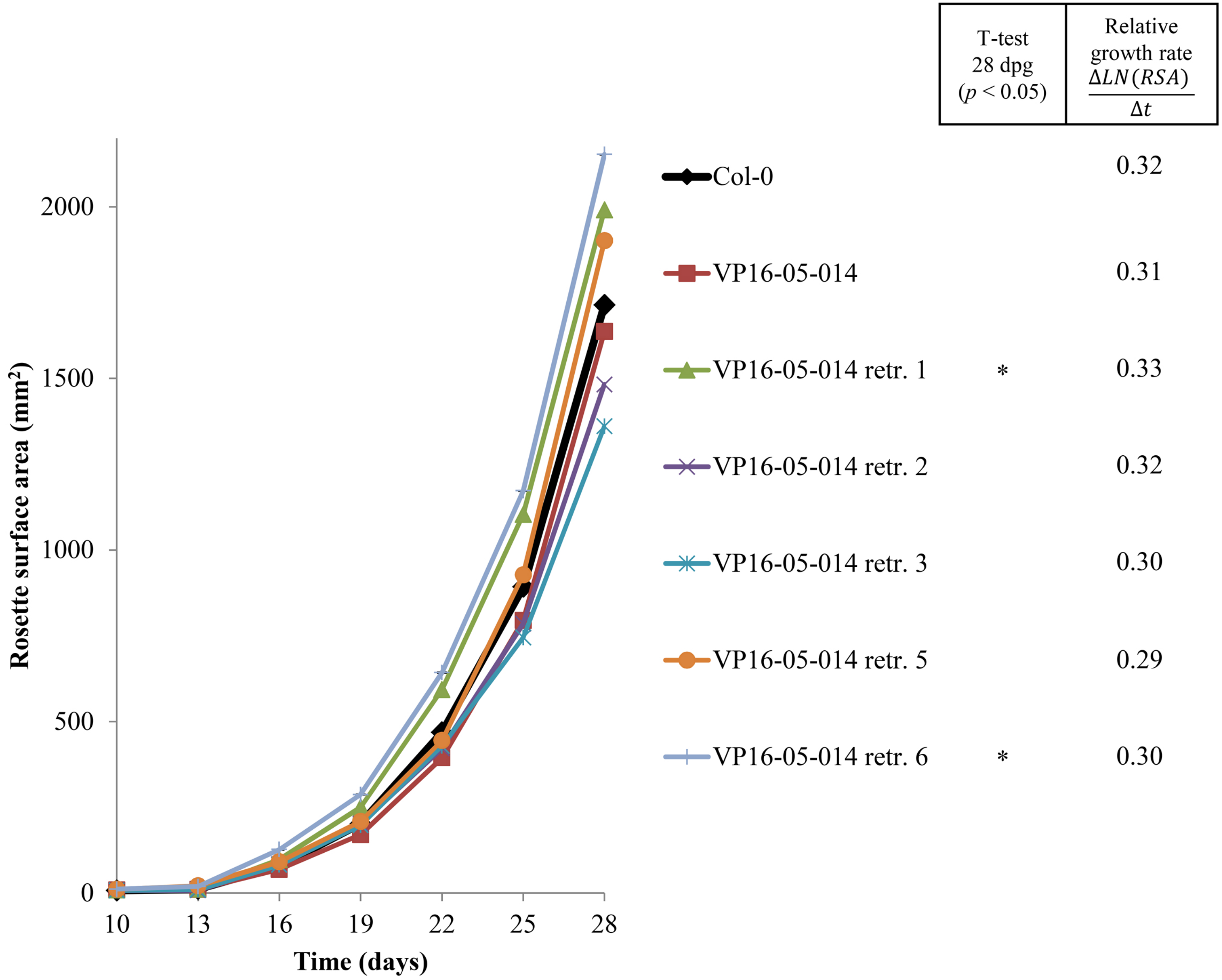

S2 Fig. Growth curves of the wild type Col-0, VP16-05-014 (T3; segregating) and retransformants reconstituted from VP16-05-014 (T2; segregating) (n=36 for Col-0, n=18 for the other genotypes). In this experiment we were not able to reproduce the increase in RSA of VP16-05-014. Significant differences with Col-0 at 28 dpg are indicated by an * ($p < 0.05$). For each genotype the average relative growth rate is provided.
