# Supplementary material for: Enhancement of Arabidopsis growth characteristics using genome interrogation with artificial transcription factors
Source: PLoS One. 2017 Mar 30;12(3):e0174236. doi: 10.1371/journal.pone.0174236 (PMC5373528; doi:10.1371/journal.pone.0174236)
Supplement: S3 Fig — The fresh and dry weights of plants of the indicated genotypes was calculated in terms of percentage of the average of Col-0. Error bars represent SEM values (n = 36 for Col-0, n = 18 for the other genotypes). Significant differences with Col-0 are indicated by an * (p < 0.05). In this experiment we were not able to reproduce the increase in biomass of VP16-05-014. (PDF) [file pone.0174236.s003.pdf]

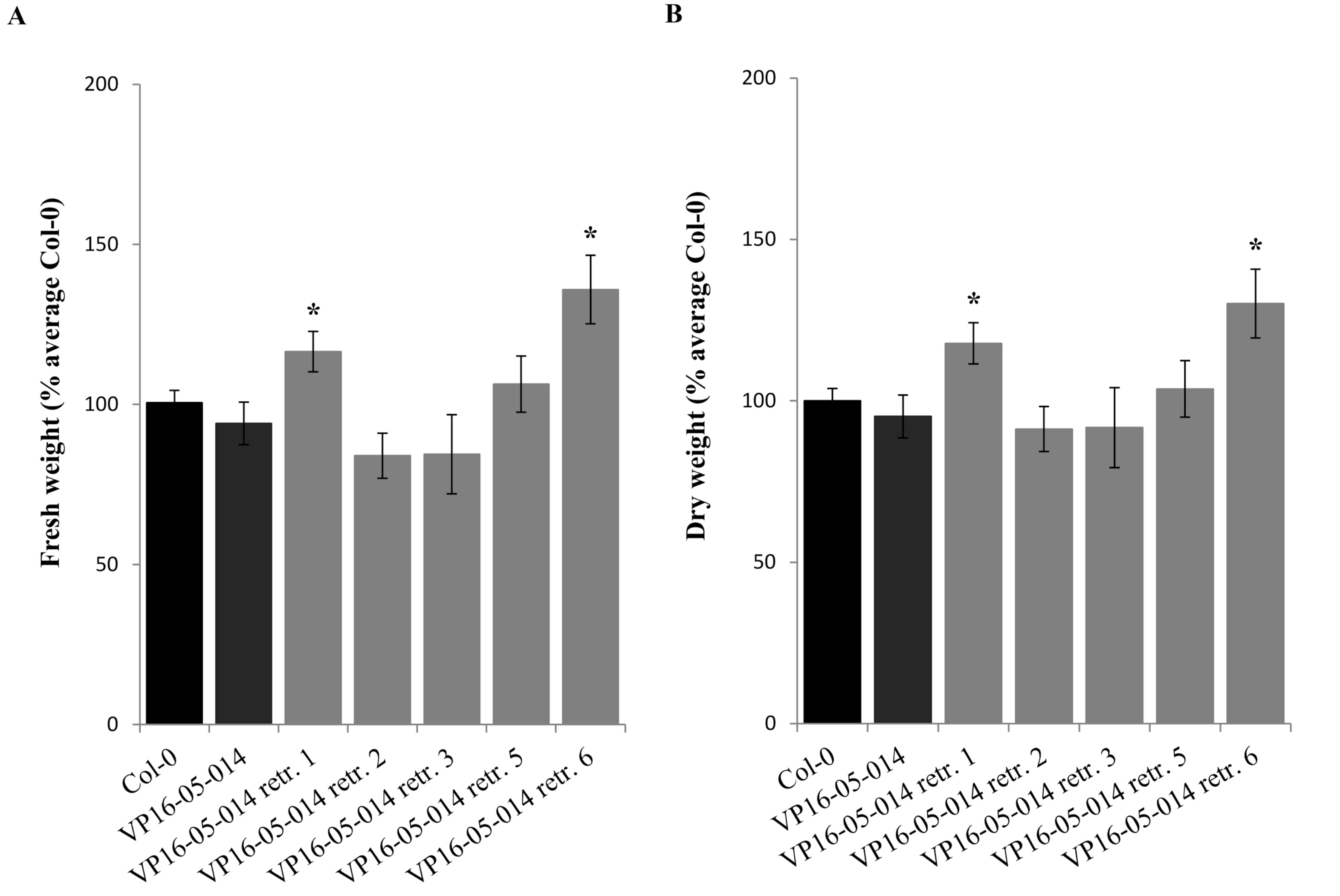

S3 Fig. Quantification of the relative fresh weight (**A**) and dry weight (**B**) of wild type Col-0 plants, VP16-05-014 plants (T3; segregating) and retransformant plants reconstituted from VP16-05-014 (T2; segregating) compared to the wild type Col-0 (28 dpg). The fresh and dry weights of plants of the indicated genotypes was calculated in terms of percentage of the average of Col-0. Error bars represent SEM values (n=36 for Col-0, n=18 for the other genotypes). Significant differences with Col-0 are indicated by an * ($p < 0.05$). In this experiment we were not able to reproduce the increase in biomass of VP16-05-014.
