# Supplementary material for: Enhancement of Arabidopsis growth characteristics using genome interrogation with artificial transcription factors
Source: PLoS One. 2017 Mar 30;12(3):e0174236. doi: 10.1371/journal.pone.0174236 (PMC5373528; doi:10.1371/journal.pone.0174236)
Supplement: S4 Fig — Significant differences with Col-0 at 27 dpg are indicated by an * (p < 0.05). For each genotype the average relative growth rate is provided. For a selection of the genotypes the average normalized expression values of the 3F-VP16 construct as determined by RT-qPCR analysis is provided. (PDF) [file pone.0174236.s004.pdf]

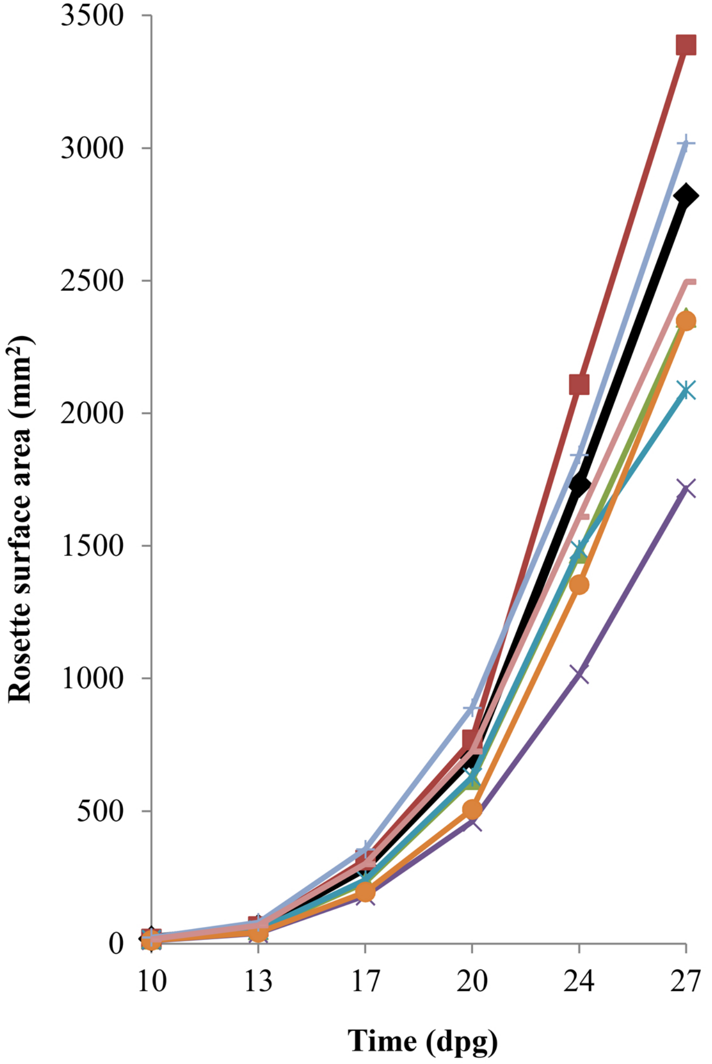

	T-test 27 dpg ($p < 0.05$)	Relative growth rate $\frac{\Delta LN(RSA)}{\Delta t}$	Average normalized expression of 3F-VP16 construct
Col-0		0.30	0.00
VP16-02-003	*	0.31	0.18
VP16-02-003 p35S retr. 1		0.30	0.60
VP16-02-003 p35S retr. 2	*	0.29	0.63
VP16-02-003 p35S retr. 3	*	0.29	1.31
VP16-02-003 p35S retr. 4	*	0.31	1.52
VP16-02-003 p35S retr. 5		0.29	
VP16-02-003 p35S retr. 6	*	0.30	

S4 Fig. Growth curves of the wild type Col-0, VP16-02-003 (T3) and retransformants reconstituted from VP16-02-003 in the binary vector construct p35S-VP16-Kana (T2; segregating) (n=84 for Col-0, n=11-18 for the transgenic lines). Significant differences with Col-0 at 27 dpg are indicated by an * ($p < 0.05$). For each genotype the average relative growth rate is provided. For a selection of the genotypes the average normalized expression values of the 3F-VP16 construct as determined by RT-qPCR analysis is provided.
