# Supplementary material for: Enhancement of Arabidopsis growth characteristics using genome interrogation with artificial transcription factors
Source: PLoS One. 2017 Mar 30;12(3):e0174236. doi: 10.1371/journal.pone.0174236 (PMC5373528; doi:10.1371/journal.pone.0174236)
Supplement: S1 Table — ‘Background’ refers to RNA expression data derived from the pool of lines expressing 3F-EAR fusions similar to the specific 3F-EAR fusion expressed in the selected lines, but without a noticeable increase in RSA. ‘Overlap’ refers to the DEGs shared in a column. (PDF) [file pone.0174236.s006.pdf]

S1 Table. Overview of differentially expressed genes (DEGs) compared to the wild type Col-0 in the RNA sequencing data sets of the indicated 3F-EAR transgenic lines ($p < 0.0001$). 'Background' refers to RNA expression data derived from the pool of lines expressing 3F- EAR fusions similar to the specific 3F-EAR fusion expressed in the selected lines, but without a noticeable increase in RSA. 'Overlap' refers to the DEGs shared in a column.

	**DEGs without subtraction of background**			**DEGs with subtraction of background**		
**Genotype**	**Total**	**Down-regulated**	**Up-regulated**	**Total**	**Down-regulated**	**Up-regulated**
**EAR-13-068**	519	271	248	261	93	168
**EAR-15-025**	283	162	121	121	38	83
**EAR-15-053**	372	215	157	180	74	106
**EAR pool 13 background**	404	266	138	-	-	-
**EAR pool 15 background**	306	208	98	-	-	-
**Overlap**	116	92	24	10	0	10
