# Supplementary material for: Enhancement of Arabidopsis growth characteristics using genome interrogation with artificial transcription factors
Source: PLoS One. 2017 Mar 30;12(3):e0174236. doi: 10.1371/journal.pone.0174236 (PMC5373528; doi:10.1371/journal.pone.0174236)
Supplement: S3 Table — (PDF) [file pone.0174236.s008.pdf]

S3 Table. Overview of significantly enriched GO categories ($p < 0.05$) found for the 104 upregulated (**Up**) and 53 downregulated (**Down**) DEGs compared to the wild type Col-0 that are shared in the RNA sequencing data sets of the three larger 3F-EAR transgenic lines, EAR-13-68, EAR-15-025 and EAR-15-053.

**Up**						
**GO biological process**	**GO term**	**Total number of genes in GO term**	**Number of genes**	**Expected value**	**Fold Enrichment**	**P-value**
xanthophyll metabolic process	GO:0016122	13	3	0.03	> 100	5.08E-03
regulation of photosynthesis, light reaction	GO:0042548	19	3	0.04	81.49	1.57E-02
regulation of generation of precursor metabolites and energy	GO:0043467	21	3	0.04	73.73	2.12E-02
carotenoid metabolic process	GO:0016116	35	4	0.07	58.98	1.54E-03
tetraterpenoid metabolic process	GO:0016108	35	4	0.07	58.98	1.54E-03
carotenoid biosynthetic process	GO:0016117	27	3	0.05	57.34	4.47E-02
tetraterpenoid biosynthetic process	GO:0016109	27	3	0.05	57.34	4.47E-02
regulation of photosynthesis	GO:0010109	27	3	0.05	57.34	4.47E-02
response to high light intensity	GO:0009644	56	4	0.11	36.86	9.77E-03
response to blue light	GO:0009637	57	4	0.11	36.22	1.05E-02
photosynthesis, light reaction	GO:0019684	77	4	0.15	26.81	3.39E-02
response to light intensity	GO:0009642	99	5	0.19	26.06	3.17E-03
circadian rhythm	GO:0007623	82	4	0.16	25.17	4.33E-02
isoprenoid metabolic process	GO:0006720	126	5	0.24	20.48	1.02E-02
photosynthesis	GO:0015979	161	5	0.31	16.03	3.30E-02
response to light stimulus	GO:0009416	547	15	1.06	14.15	2.05E-10
response to radiation	GO:0009314	568	15	1.1	13.63	3.51E-10
response to abiotic stimulus	GO:0009628	1468	20	2.84	7.03	2.91E-09
response to inorganic substance	GO:0010035	682	9	1.32	6.81	1.26E-02
response to acid chemical	GO:0001101	860	10	1.67	6	1.10E-02
response to stimulus	GO:0050896	4612	24	8.94	2.69	2.74E-03
**Down**						
**GO biological process**	**GO term**	**Total number of genes in GO term**	**Number of genes**	**Expected value**	**Fold Enrichment**	**P-value**
regulation of circadian rhythm	GO:0042752	31	4	0.12	33.94	1.43E-02
circadian rhythm	GO:0007623	82	6	0.31	19.24	1.76E-03
rhythmic process	GO:0048511	98	7	0.37	18.79	2.44E-04
