# Supplementary material for: Enhancement of Arabidopsis growth characteristics using genome interrogation with artificial transcription factors
Source: PLoS One. 2017 Mar 30;12(3):e0174236. doi: 10.1371/journal.pone.0174236 (PMC5373528; doi:10.1371/journal.pone.0174236)
Supplement: S4 Table — (PDF) [file pone.0174236.s009.pdf]

S4 Table. Overview of the 116 differentially expressed genes (DEGs) compared to the wild type Col-0 that are shared in all RNA sequencing data sets derived from 3F-EAR transgenic lines and background pools ($p < 0.0001$).

Locus ID	Gene name	Annotation	Up or downregulated
AT1G01060	LHY	Protein LHY	Up
AT1G06430	FTSH8	ATP-dependent zinc metalloprotease FTSH 8, chloroplastic	Up
AT1G06460	ACD32.1	alpha-crystallin domain 32.1	Down
AT1G06570	PDS1	phytoene desaturation 1	Up
AT1G07050		CCT motif family protein	Down
AT1G07180	NDA1	Internal alternative NAD(P)H-ubiquinone oxidoreductase A1, mitochondrial	Up
AT1G11210		Protein of unknown function (DUF761)	Down
AT1G12710	P2A12	F-box protein PP2-A12	Down
AT1G13270	MAP1B	Methionine aminopeptidase 1B, chloroplastic	Down
AT1G17665		unknown protein	Down
AT1G18710	AtMYB47	myb domain protein 47	Down
AT1G20020	LFNR2	Ferredoxin--NADP reductase, leaf isozyme 2, chloroplastic	Down
AT1G20030		Pathogenesis-related thaumatin superfamily protein	Down
AT1G20510	4CLL5	4-coumarate--CoA ligase-like 5	Down
AT1G20693	HMGB2	High mobility group B protein 2	Down
AT1G20696	HMGB3	High mobility group B protein 3	Down
AT1G21600	PTAC6	plastid transcriptionally active 6	Down
AT1G21680		DPP6 N-terminal domain-like protein	Down
AT1G22770	GI	Protein GIGANTEA	Down
AT1G33260		Probable receptor-like protein kinase At1g33260	Up
AT1G48330		unknown protein	Down
AT1G49720	ABF1	ABSCISIC ACID-INSENSITIVE 5-like protein 4	Down
AT1G51610	MTPC4	Metal tolerance protein C4	Down
AT1G53035		unknown protein	Down
AT1G56300		Chaperone DnaJ-domain superfamily protein	Down
AT1G62430	CDS1	Phosphatidate cytidylyltransferase 1	Down
AT1G64760		Glucan endo-1,3-beta-glucosidase 8	Down
AT1G67660		Restriction endonuclease, type II-like superfamily protein	Down
AT1G69730	WAKL9	Wall-associated receptor kinase-like 9	Up
AT1G70420		Protein of unknown function (DUF1645)	Down
AT1G75100	JAC1	J domain-containing protein required for chloroplast accumulation response 1	Up
AT1G76590		PLATZ transcription factor family protein	Down

AT1G80480	PTAC17	plastid transcriptionally active 17	Down
AT2G02100	PDF2.2	Defensin-like protein 2	Down
AT2G15970	COR413PM1	Cold-regulated 413 plasma membrane protein 1	Down
AT2G16365		F-box protein At2g16365	Down
AT2G21130	CYP19-2	Peptidyl-prolyl cis-trans isomerase CYP19-2	Down
AT2G21660	RBG7	Glycine-rich RNA-binding protein 7	Down
AT2G22450	RIBA2	Monofunctional riboflavin biosynthesis protein RIBA 2, chloroplastic	Down
AT2G24100		unknown protein	Up
AT2G28190	CSD2	Superoxide dismutase [Cu-Zn] 2, chloroplastic	Up
AT2G28900	OEP161	Outer envelope pore protein 16-1, chloroplastic	Down
AT2G29630	THIC	Phosphomethylpyrimidine synthase, chloroplastic	Down
AT2G36390	SBE2.1	1,4-alpha-glucan-branching enzyme 2-1, chloroplastic/amyloplastic	Down
AT2G37220	CP29B	RNA-binding protein CP29B, chloroplastic	Down
AT2G38465		unknown protein	Down
AT2G38550	FAX3	Protein FATTY ACID EXPORT 3, chloroplastic	Down
AT2G39900	WLIN2A	LIM domain-containing protein WLIM2a	Down
AT2G39920		Uncharacterized protein At2g39920	Down
AT2G40080	ELF4	Protein EARLY FLOWERING 4	Down
AT2G40100	LHCB4.3	Chlorophyll a-b binding protein CP29.3, chloroplastic	Up
AT2G42530	COR15B	Protein COLD-REGULATED 15B, chloroplastic	Down
AT2G43535	ATTI4	Defensin-like protein 196	Down
AT2G47800	ABCC4	ABC transporter C family member 4	Up
AT2G47890	COL13	Zinc finger protein CONSTANS-LIKE 13	Down
AT3G04550	RAF2	Rubisco accumulation factor 2, chloroplastic	Down
AT3G05880	RCI2A	Hydrophobic protein RCI2A	Down
AT3G07650	COL9	Zinc finger protein CONSTANS-LIKE 9	Down
AT3G07700		Protein kinase superfamily protein	Up
AT3G09600	RVE8	Protein REVEILLE 8	Up
AT3G10410	SCPL49	Serine carboxypeptidase-like 49	Down
AT3G10420	SPD1	P-loop containing nucleoside triphosphate hydrolases superfamily protein	Up
AT3G12320	T2E22.34	unknown protein	Up
AT3G17800		Protein of unknown function (DUF760)	Up
AT3G29240		Protein of unknown function (DUF179)	Up
AT3G46640	LUX	Transcription factor LUX	Down
AT3G47500	CDF3	Cyclic dof factor 3	Up
AT3G47860	CHL	chloroplastic lipocalin	Down
AT3G53530	NAKR3	Chloroplast-targeted copper chaperone protein	Down

AT3G63160		FUNCTIONS IN: molecular_function unknown	Down
AT4G04330		Chaperonin-like RbcX protein	Down
AT4G08290		WAT1-related protein At4g08290	Up
AT4G11600	GPX6	Probable phospholipid hydroperoxide glutathione peroxidase 6, mitochondrial	Down
AT4G12900		Gamma interferon responsive lysosomal thiol (GILT) reductase family protein	Down
AT4G13010		Putative quinone-oxidoreductase homolog, chloroplastic	Up
AT4G13250	NYC1	Probable chlorophyll(ide) b reductase NYC1, chloroplastic	Down
AT4G15530	PPDK	Pyruvate, phosphate dikinase 1, chloroplastic	Up
AT4G16146		cAMP-regulated phosphoprotein 19-related protein	Down
AT4G21215		unknown protein	Down
AT4G26670	TIM22-2	Mitochondrial import inner membrane translocase subunit TIM22-2	Down
AT4G27130		Protein translation factor SUI1 homolog 1	Down
AT4G29610	CDA6	Cytidine deaminase 6	Down
AT4G30650		UPF0057 membrane protein At4g30650	Down
AT4G30660		UPF0057 membrane protein At4g30660	Down
AT4G32340		Tetratricopeptide repeat (TPR)-like superfamily protein	Down
AT4G33467		unknown protein	Down
AT4G33490		Eukaryotic aspartyl protease family protein	Down
AT4G33700	CBSDUF6	DUF21 domain-containing protein At4g33700	Down
AT4G33980		BEST Arabidopsis thaliana protein match is: cold regulated gene 27 (TAIR:AT5G42900.2)	Down
AT4G34900	XDH2	Xanthine dehydrogenase 2	Down
AT4G39260	RBG8	Glycine-rich RNA-binding protein 8	Down
AT5G03470	B'ALPHA	Serine/threonine protein phosphatase 2A 57 kDa regulatory subunit B' alpha isoform	Down
AT5G06530	ABCG22	ABC transporter G family member 22	Up
AT5G06980		unknown protein	Up
AT5G11150	VAMP713	Vesicle-associated membrane protein 713	Down
AT5G14550		Core-2/I-branching beta-1,6-N-acetylglucosaminyltransferase family protein	Down
AT5G15230	GASA4	Gibberellin-regulated protein 4	Down
AT5G18540		unknown protein	Down
AT5G20630	GER3	Germin-like protein subfamily 3 member 3	Down
AT5G23240		DNAJ heat shock N-terminal domain-containing protein	Down
AT5G24060		Pentatricopeptide repeat (PPR) superfamily protein	Down
AT5G26570	GWD3	Phosphoglucan, water dikinase, chloroplastic	Down
AT5G39410		Probable mitochondrial saccharopine	Down

		dehydrogenase-like oxidoreductase At5g39410	
AT5G47240	NUDT8	Nudix hydrolase 8	Down
AT5G48250	COL10	Zinc finger protein CONSTANS-LIKE 10	Down
AT5G49015		Expressed protein	Down
AT5G50450		F-box protein At5g50450	Down
AT5G53370	PME61	Probable pectinesterase/pectinesterase inhibitor 61	Up
AT5G57110	ACA8	Calcium-transporting ATPase 8, plasma membrane-type	Down
AT5G60100	APRR3	Two-component response regulator-like APRR3	Down
AT5G60540	PDX2	Probable pyridoxal 5'-phosphate synthase subunit PDX2	Down
AT5G61380	APRR1	Two-component response regulator-like APRR1	Down
AT5G62360		Plant invertase/pectin methylesterase inhibitor superfamily protein	Down
AT5G62720		Integral membrane HPP family protein	Down
AT5G63420	emb2746	RNA-metabolising metallo-beta-lactamase family protein	Down
AT5G64860	DPE1	4-alpha-glucanotransferase DPE1, chloroplastic/amyloplastic	Down
