# Supplementary material for: Enhancement of Arabidopsis growth characteristics using genome interrogation with artificial transcription factors
Source: PLoS One. 2017 Mar 30;12(3):e0174236. doi: 10.1371/journal.pone.0174236 (PMC5373528; doi:10.1371/journal.pone.0174236)
Supplement: S5 Table — (PDF) [file pone.0174236.s010.pdf]

S5 Table. Overview of significantly enriched GO categories ($p < 0.05$) found for the 24 upregulated (**Up**) and 92 downregulated (**Down**) DEGs compared to the wild type Col-0 that are shared in the RNA sequencing data sets derived from 3F-EAR transgenic lines and background pools.

**Up**						
**GO biological process**	**GO term**	**Total number of genes in GO term**	**Number of genes**	**Expected value**	**Fold Enrichment**	**P-value**
regulation of photosynthesis, light reaction	GO:0042548	14	3	0.03	> 100	5.82E-03
regulation of generation of precursor metabolites and energy	GO:0043467	15	3	0.03	> 100	7.15E-03
photosynthesis, light harvesting	GO:0009765	20	3	0.04	76.97	1.68E-02
regulation of photosynthesis	GO:0010109	21	3	0.04	73.31	1.95E-02
response to blue light	GO:0009637	44	4	0.09	46.65	3.49E-03
response to high light intensity	GO:0009644	45	4	0.09	45.61	3.82E-03
photosynthesis, light reaction	GO:0019684	60	4	0.12	34.21	1.18E-02
response to light intensity	GO:0009642	76	5	0.15	33.76	8.10E-04
cellular response to light stimulus	GO:0071482	74	4	0.14	27.74	2.68E-02
cellular response to radiation	GO:0071478	79	4	0.15	25.98	3.45E-02
photosynthesis	GO:0015979	133	5	0.26	19.29	1.22E-02
response to light stimulus	GO:0009416	422	13	0.82	15.81	2.57E-09
response to radiation	GO:0009314	442	13	0.86	15.09	4.56E-09
response to abiotic stimulus	GO:0009628	1121	19	2.18	8.7	2.59E-10
response to inorganic substance	GO:0010035	536	8	1.04	7.66	1.69E-02
response to stimulus	GO:0050896	3894	22	7.59	2.9	2.32E-03
**Down**						
**GO biological process**	**GO term**	**Total number of genes in GO term**	**Number of genes**	**Expected value**	**Fold Enrichment**	**P-value**
rhythmic process	GO:0048511	76	5	0.29	17.21	2.31E-02
