# Supplementary material for: Enhancement of Arabidopsis growth characteristics using genome interrogation with artificial transcription factors
Source: PLoS One. 2017 Mar 30;12(3):e0174236. doi: 10.1371/journal.pone.0174236 (PMC5373528; doi:10.1371/journal.pone.0174236)
Supplement: S6 Table — (PDF) [file pone.0174236.s011.pdf]

S6 Table. Overview of significantly enriched GO categories ($p < 0.05$) found for the selection of 10 out of 157 DEGs compared to the wild type Col-0 that are shared in the RNA sequencing data sets of the three larger 3F-EAR transgenic lines, but are not found in the transcriptomes of background pools.

**GO biological process**	**GO term**	**Total number of genes in GO term**	**Number of genes**		**Expected value**	**Fold Enrichment**	**P-value**	**Gene IDs**
xanthophyll biosynthetic process	GO:0016123	6	2	33.3%	0	> 100	445E-03	AT5G52570<br>AT5G67030
xanthophyll metabolic process	GO:0016122	13	3	23.1%	0	> 100	264E-05	AT5G52570<br>AT5G67030<br>AT4G32770
carotenoid metabolic process	GO:0016116	35	3	8.6%	0.01	> 100	514E-04	AT5G52570<br>AT5G67030<br>AT4G32770
tetraterpenoid metabolic process	GO:0016108	35	3	8.6%	0.01	> 100	514E-04	AT5G52570<br>AT5G67030<br>AT4G32770
terpenoid metabolic process	GO:0006721	104	3	2.9%	0.04	78.9	133E-02	AT5G52570<br>AT5G67030<br>AT4G32770
isoprenoid metabolic process	GO:0006720	126	4	3.2%	0.05	86.83	190E-04	AT5G52570<br>AT5G67030<br>AT4G34350<br>AT4G32770
cellular lipid metabolic process	GO:0044255	471	4	0.8%	0.17	23.23	350E-02	AT5G52570<br>AT5G67030<br>AT4G34350<br>AT4G32770
isoprenoid biosynthetic process	GO:0008299	115	3	2.6%	0.04	71.35	179E-02	AT5G52570<br>AT5G67030<br>AT4G34350
monocarboxylic acid metabolic process	GO:0032787	415	5	1.2%	0.15	32.95	391E-04	AT5G52570<br>AT5G67030<br>AT4G34350<br>AT4G38970<br>AT4G32770
carboxylic acid metabolic process	GO:0019752	731	5	0.7%	0.27	18.71	632E-03	AT5G52570<br>AT5G67030<br>AT4G34350<br>AT4G38970<br>AT4G32770
oxoacid metabolic process	GO:0043436	802	5	0.6%	0.29	17.05	993E-03	AT5G52570<br>AT5G67030<br>AT4G34350<br>AT4G38970<br>AT4G32770
organic acid metabolic process	GO:0006082	900	5	0.6%	0.33	15.2	174E-02	AT5G52570<br>AT5G67030<br>AT4G34350<br>AT4G38970<br>AT4G32770<br>AT1G23310
