# Supplementary material for: Enhancement of Arabidopsis growth characteristics using genome interrogation with artificial transcription factors
Source: PLoS One. 2017 Mar 30;12(3):e0174236. doi: 10.1371/journal.pone.0174236 (PMC5373528; doi:10.1371/journal.pone.0174236)
Supplement: S7 Table — ‘Background’ refers to RNA expression data derived from the pool of lines expressing 3F-VP16 fusions similar to the specific 3F-VP16 fusion expressed in the selected lines, but without a noticeable increase in RSA. ‘Overlap’ refers to the DEGs shared in a column. (PDF) [file pone.0174236.s012.pdf]

S7 Table. Overview of differentially expressed genes (DEGs) compared to the wild type Col-0 in the RNA sequencing data sets of the indicated 3F-VP16 transgenic lines ($p < 0.0001$). 'Background' refers to RNA expression data derived from the pool of lines expressing 3F- VP16 fusions similar to the specific 3F-VP16 fusion expressed in the selected lines, but without a noticeable increase in RSA. 'Overlap' refers to the DEGs shared in a column.

	**DEGs without subtraction of background**			**DEGs with subtraction of background**		
**Genotype**	**Total**	**Down-regulated**	**Up-regulated**	**Total**	**Down-regulated**	**Up-regulated**
**VP16-02- 003**	1539	723	816	1531	718	813
**VP16-05- 014**	272	103	169	271	102	169
**VP16 pool 2 background**	38	12	26	-	-	-
**VP16 pool 5 background**	12	11	1	-	-	-
**Overlap**	0	0	0	237	92	145
