# Supplementary material for: Enhancement of Arabidopsis growth characteristics using genome interrogation with artificial transcription factors
Source: PLoS One. 2017 Mar 30;12(3):e0174236. doi: 10.1371/journal.pone.0174236 (PMC5373528; doi:10.1371/journal.pone.0174236)
Supplement: S8 Table — The 2 DEGs that were also found in the transcriptomes of background pools are shaded. (PDF) [file pone.0174236.s013.pdf]

**S8 Table.** Overview of the 239 differentially expressed genes (DEGs) compared to the wild type Col-0 that are shared in the RNA sequencing data sets of the two larger 3F-VP16 transgenic lines, VP16-02-003 and VP16-005-014 ($p < 0.0001$). The 2 DEGs that were also found in the transcriptomes of background pools are shaded in grey.

| Locus ID | Gene name | Annotation | Up or downregulated |
|---|---|---|---|
| AT1G01060 | LHY | Protein LHY | Up |
| AT1G01560 | MPK11 | Mitogen-activated protein kinase 11 | Up |
| AT1G02930 | GSTF6 | Glutathione S-transferase F6 | Up |
| AT1G03290 | | unknown protein | Up |
| AT1G03400 | | 1-aminocyclopropane-1-carboxylate oxidase homolog 4 | Up |
| AT1G05010 | ACO4 | 1-aminocyclopropane-1-carboxylate oxidase 4 | Up |
| AT1G06410 | TPS7 | Probable alpha,alpha-trehalose-phosphate synthase [UDP-forming] 7 | Up |
| AT1G07050 | | CCT motif family protein | Down |
| AT1G08450 | CRT3 | Calreticulin-3 | Up |
| AT1G09250 | BHLH149 | Transcription factor bHLH149 | Up |
| AT1G09430 | ACLA-3 | ATP-citrate synthase alpha chain protein 3 | Up |
| AT1G10657 | | Plant protein 1589 of unknown function | Down |
| AT1G11670 | | MATE efflux family protein | Down |
| AT1G12090 | ELP | extensin-like protein | Down |
| AT1G12360 | KEU | SNARE-interacting protein KEULE | Up |
| AT1G13000 | | Protein of unknown function (DUF707) | Up |
| AT1G13650 | | BEST Arabidopsis thaliana protein match is: 18S pre-ribosomal assembly protein gar2-related (TAIR:AT2G03810.4) | Down |
| AT1G13930 | | Involved in response to salt stress. Knockout mutants are hypersensitive to salt stress. | Down |
| AT1G14280 | PKS2 | Protein PHYTOCHROME KINASE SUBSTRATE 2 | Up |
| AT1G14920 | GAI | DELLA protein GAI | Up |
| AT1G15140 | | FAD/NAD(P)-binding oxidoreductase | Down |
| AT1G15670 | | F-box/kelch-repeat protein At1g15670 | Up |
| AT1G16260 | WAKL8 | Wall-associated receptor kinase-like 8 | Up |
| AT1G16510 | SAUR41 | Auxin-responsive protein SAUR41 | Up |
| AT1G18400 | BEE1 | Transcription factor BEE 1 | Up |
| AT1G18710 | AtMYB47 | myb domain protein 47 | Down |
| AT1G20020 | LFNR2 | Ferredoxin--NADP reductase, leaf isozyme 2, chloroplastic | Down |
| AT1G20030 | | Pathogenesis-related thaumatin superfamily protein | Down |
| AT1G20510 | 4CLL5 | 4-coumarate--CoA ligase-like 5 | Down |
| AT1G20780 | PUB44 | U-box domain-containing protein 44 | Up |
| AT1G21050 | | Protein of unknown function, DUF617 | Up |
| AT1G21250 | WAK1 | Wall-associated receptor kinase 1 | Up |
| AT1G21270 | WAK2 | Wall-associated receptor kinase 2 | Up |

| | | | |
|---|---|---|---|
| AT1G22530 | PATL2 | Patellin-2 | Up |
| AT1G22770 | GI | Protein GIGANTEA | Down |
| AT1G22882 | | Galactose-binding protein | Up |
| AT1G23090 | SULTR3 | | Up |
| AT1G24100 | UGT74B1 | UDP-glycosyltransferase 74B1 | Down |
| AT1G26380 | | FAD-binding Berberine family protein | Up |
| AT1G26420 | | FAD-binding Berberine family protein | Up |
| AT1G31690 | | Copper amine oxidase family protein | Down |
| AT1G32640 | MYC2 | Transcription factor MYC2 | Down |
| AT1G33170 | | Probable methyltransferase PMT18 | Down |
| AT1G34300 | | G-type lectin S-receptor-like serine/threonine-protein kinase At1g34300 | Up |
| AT1G43700 | VIP1 | Transcription factor VIP1 | Up |
| AT1G45010 | | TRAM, LAG1 and CLN8 (TLC) lipid-sensing domain containing protein | Down |
| AT1G48330 | | unknown protein | Down |
| AT1G51800 | | Leucine-rich repeat protein kinase family protein | Up |
| AT1G53570 | MAP3KA | mitogen-activated protein kinase kinase kinase 3 | Up |
| AT1G55260 | | Bifunctional inhibitor/lipid-transfer protein/seed storage 2S albumin superfamily protein | Down |
| AT1G56150 | SAUR71 | Auxin-responsive protein SAUR71 | Up |
| AT1G58225 | | unknown protein | Up |
| AT1G59710 | | Protein of unknown function (DUF569) | Up |
| AT1G61890 | | MATE efflux family protein | Down |
| AT1G62430 | CDS1 | Phosphatidate cytidylyltransferase 1 | Down |
| AT1G63900 | DAL1 | E3 Ubiquitin ligase family protein | Up |
| AT1G64400 | LACS3 | Long chain acyl-CoA synthetase 3 | Up |
| AT1G64740 | TUBA1 | Tubulin alpha-1 chain | Up |
| AT1G64760 | | Glucan endo-1,3-beta-glucosidase 8 | Down |
| AT1G65790 | SD17 | Receptor-like serine/threonine-protein kinase SD1-7 | Up |
| AT1G69730 | WAKL9 | Wall-associated receptor kinase-like 9 | Up |
| AT1G70700 | TIFY7 | Protein TIFY 7 | Down |
| AT1G70940 | PIN3 | Auxin efflux carrier component 3 | Up |
| AT1G72520 | LOX4 | Lipoxygenase 4, chloroplastic | Down |
| AT1G73080 | PEPR1 | Leucine-rich repeat receptor-like protein kinase PEPR1 | Down |
| AT1G74100 | SOT16 | Cytosolic sulfotransferase 16 | Down |
| AT1G74360 | | Probable LRR receptor-like serine/threonine-protein kinase At1g74360 | Up |
| AT1G76970 | | Target of Myb protein 1 | Up |
| AT1G80480 | PTAC17 | plastid transcriptionally active 17 | Down |
| AT2G01890 | PAP8 | Purple acid phosphatase 8 | Down |
| AT2G14750 | APK1 | Adenylyl-sulfate kinase 1, chloroplastic | Down |
| AT2G17120 | LYM2 | LysM domain-containing GPI-anchored protein 2 | Up |

| | | | |
|---|---|---|---|
| AT2G21130 | CYP19-2 | Peptidyl-prolyl cis-trans isomerase CYP19-2 | Down |
| AT2G22330 | CYP79B3 | Tryptophan N-monooxygenase 2 | Down |
| AT2G23170 | GH3.3 | Indole-3-acetic acid-amido synthetase GH3.3 | Up |
| AT2G23670 | YCF37 | homolog of Synechocystis YCF37 | Down |
| AT2G24100 | | unknown protein | Up |
| AT2G24360 | | Protein kinase superfamily protein | Up |
| AT2G25450 | GSL-OH | Probable 2-oxoacid dependent dioxygenase | Down |
| AT2G25510 | | unknown protein | Up |
| AT2G28900 | OEP161 | Outer envelope pore protein 16-1, chloroplastic | Down |
| AT2G29110 | GLR2.8 | Glutamate receptor 2.8 | Up |
| AT2G29300 | | Tropinone reductase homolog At2g29300 | Down |
| AT2G29450 | GSTU5 | Glutathione S-transferase U5 | Down |
| AT2G29630 | THIC | Phosphomethylpyrimidine synthase, chloroplastic | Down |
| AT2G31020 | ORP1A | Oxysterol-binding protein-related protein 1A | Up |
| AT2G32230 | PRORP1 | Proteinaceous RNase P 1, chloroplastic/mitochondrial | Down |
| AT2G34600 | TIFY 5B | Protein TIFY 5B | Down |
| AT2G36390 | SBE2.1 | 1,4-alpha-glucan-branching enzyme 2-1, chloroplastic/amyloplastic | Down |
| AT2G38310 | PYL4 | Abscisic acid receptor PYL4 | Up |
| AT2G39920 | | Uncharacterized protein At2g39920 | Down |
| AT2G40080 | ELF4 | Protein EARLY FLOWERING 4 | Down |
| AT2G40940 | ERS1 | Ethylene response sensor 1 | Up |
| AT2G41110 | CAM2 | Calmodulin-5 | Up |
| AT2G42530 | COR15B | Protein COLD-REGULATED 15B, chloroplastic | Down |
| AT2G42580 | TTL3 | Inactive TPR repeat-containing thioredoxin TTL3 | Up |
| AT2G37440 | | DNAse I-like superfamily protein | Up |
| AT2G44230 | | Plant protein of unknown function (DUF946) | Down |
| AT2G45560 | CYP76C1 | Cytochrome P450 76C1 | Down |
| AT2G46270 | GBF3 | G-box-binding factor 3 | Up |
| AT2G46330 | AGP16 | Arabinogalactan peptide 16 | Up |
| AT2G46430 | CNGC3 | Probable cyclic nucleotide-gated ion channel 3 | Up |
| AT2G47440 | | Tetratricopeptide repeat (TPR)-like superfamily protein | Up |
| AT2G48030 | | DNAse I-like superfamily protein | Up |
| AT3G01290 | HIR3 | Hypersensitive-induced response protein 3 | Up |
| AT3G07780 | OBE1 | Protein OBERON 1 | Up |
| AT3G09940 | MDHAR | Probable monodehydroascorbate reductase, cytoplasmic isoform 1 | Up |
| AT3G10410 | SCPL49 | Serine carboxypeptidase-like 49 | Down |
| AT3G16000 | MFP1 | MAR-binding filament-like protein 1 | Down |
| AT3G16030 | CES101 | G-type lectin S-receptor-like serine/threonine-protein kinase CES101 | Up |
| AT3G16250 | PNSB3 | Photosynthetic NDH subunit of subcomplex B 3, | Down |

| | | | |
|---|---|---|---|
| | | chloroplastic | |
| AT3G17330 | ECT6 | evolutionarily conserved C-terminal region 6 | Down |
| AT3G18080 | BGLU44 | Beta-glucosidase 44 | Down |
| AT3G19850 | | BTB/POZ domain-containing protein At3g19850 | Up |
| AT3G19930 | STP4 | Sugar transport protein 4 | Up |
| AT3G21330 | BHLH87 | Transcription factor bHLH87 | Up |
| AT3G22840 | ELIP1 | Early light-induced protein 1, chloroplastic | Up |
| AT3G26210 | CYP71B23 | Cytochrome P450 71B23 | Up |
| AT3G26220 | CYP71B3 | Cytochrome P450 71B3 | Up |
| AT3G26830 | CYP71B15 | Bifunctional dihydrocamalexate synthase/camalexin synthase | Up |
| AT3G28070 | | WAT1-related protein At3g28070 | Down |
| AT3G28130 | | WAT1-related protein At3g28130 | Down |
| AT3G30180 | CYP85A2 | Cytochrome P450 85A2 | Up |
| AT3G44990 | XTH31 | Xyloglucan endotransglucosylase/hydrolase protein 31 | Down |
| AT3G45620 | | Transducin/WD40 repeat-like superfamily protein | Up |
| AT3G46510 | PUB13 | U-box domain-containing protein 13 | Up |
| AT3G47250 | | Plant protein of unknown function (DUF247) | Up |
| AT3G47960 | NPF2.10 | Protein NRT1/ PTR FAMILY 2.10 | Down |
| AT3G48080 | EDS1B | Protein EDS1B | Up |
| AT3G51440 | SSL6 | Protein STRICTOSIDINE SYNTHASE-LIKE 6 | Up |
| AT3G51920 | CML9 | Calmodulin-like protein 9 | Up |
| AT3G52240 | | unknown protein | Up |
| AT3G53680 | | Acyl-CoA N-acyltransferase with RING/FYVE/PHD-type zinc finger domain | Down |
| AT3G55630 | FPGS3 | Folylpolyglutamate synthase | Down |
| AT3G56040 | UGP3 | UDP-glucose pyrophosphorylase 3 | Down |
| AT3G56310 | AGAL3 | Alpha-galactosidase 3 | Up |
| AT3G56880 | | VQ motif-containing protein | Down |
| AT3G57460 | | catalytics | Up |
| AT3G57550 | GK-2 | Guanylate kinase 2 | Up |
| AT3G57800 | BHLH60 | Transcription factor bHLH60 | Up |
| AT3G58640 | | Mitogen activated protein kinase kinase kinase-related | Up |
| AT3G61630 | CRF6 | Ethylene-responsive transcription factor CRF6 | Up |
| AT3G62150 | ABCB21 | ABC transporter B family member 21 | Up |
| AT3G63080 | GPX5 | Probable glutathione peroxidase 5 | Up |
| AT4G00330 | CRCK2 | Calmodulin-binding receptor-like cytoplasmic kinase 2 | Up |
| AT4G02520 | GSTF2 | Glutathione S-transferase F2 | Up |
| AT4G03110 | BRN1 | RNA-binding protein BRN1 | Up |
| AT4G04840 | MSRB6 | Peptide methionine sulfoxide reductase B6 | Down |
| AT4G05320 | UBQ10 | Polyubiquitin 10 | Up |

| | | | |
|---|---|---|---|
| AT4G08470 | MAPKKK10 | MAPK/ERK kinase kinase 3 | Up |
| AT4G08930 | APRL6 | 5'-adenylylsulfate reductase-like 6 | Up |
| AT4G10970 | | unknown protein | Up |
| AT4G11360 | RHA1B | E3 ubiquitin-protein ligase RHA1B | Down |
| AT4G11890 | CRK45 | Cysteine-rich receptor-like protein kinase 45 | Up |
| AT4G12980 | | Cytochrome b561 and DOMON domain-containing protein At4g12980 | Down |
| AT4G14365 | XBAT34 | Putative E3 ubiquitin-protein ligase XBAT34 | Up |
| AT4G14680 | APS3 | ATP-sulfurylase 3, chloroplastic | Down |
| AT4G14746 | | CONTAINS InterPro DOMAIN/s: EGF-like (InterPro:IPR006210) | Up |
| AT4G15233 | | ABC-2 and Plant PDR ABC-type transporter family protein | Up |
| AT4G16146 | | cAMP-regulated phosphoprotein 19-related protein | Down |
| AT4G16740 | TPS03 | Tricyclene synthase, chloroplastic | Down |
| AT4G16880 | | Leucine-rich repeat (LRR) family protein | Down |
| AT4G16980 | | arabinogalactan-protein family | Down |
| AT4G18270 | ATTRANS 11 | Phospho-N-acetylmuramoyl-pentapeptide-transferase homolog | Down |
| AT4G21870 | HSP15.4 | 15.4 kDa class V heat shock protein | Up |
| AT4G22305 | | alpha/beta-Hydrolases superfamily protein | Up |
| AT4G23470 | | PLAC8 family protein | Up |
| AT4G23650 | CPK3 | Calcium-dependent protein kinase 3 | Up |
| AT4G23850 | LACS4 | Long chain acyl-CoA synthetase 4 | Up |
| AT4G25070 | | unknown protein | Up |
| AT4G25110 | AMC2 | Metacaspase-2 | Up |
| AT4G26070 | MKK1 | Mitogen-activated protein kinase kinase 1 | Up |
| AT4G26530 | | Aldolase superfamily protein | Down |
| AT4G29310 | | Protein of unknown function (DUF1005) | Up |
| AT4G29610 | CDA6 | Cytidine deaminase 6 | Down |
| AT4G30270 | XTH24 | Xyloglucan endotransglucosylase/hydrolase protein 24 | Up |
| AT4G30530 | | Class I glutamine amidotransferase-like superfamily protein | Down |
| AT4G30650 | | UPF0057 membrane protein At4g30650 | Down |
| AT4G30660 | | UPF0057 membrane protein At4g30660 | Down |
| AT4G31170 | | Protein kinase superfamily protein | Up |
| AT4G31500 | CYP83B1 | Cytochrome P450 83B1 | Down |
| AT4G31800 | WRKY18 | WRKY transcription factor 18 | Down |
| AT4G32040 | KNAT5 | Homeobox protein knotted-1-like 5 | Up |
| AT4G32440 | | Plant Tudor-like RNA-binding protein | Up |
| AT4G32480 | | Protein of unknown function (DUF506) | Down |
| AT4G33565 | | RING/U-box superfamily protein | Up |
| AT4G34750 | | SAUR-like auxin-responsive protein family | Up |

| | | | |
|---|---|---|---|
| AT4G34760 | | SAUR-like auxin-responsive protein family | Up |
| AT4G35780 | | ACT-like protein tyrosine kinase family protein | Up |
| AT4G36210 | | Protein of unknown function (DUF726) | Up |
| AT4G39260 | RBG8 | Glycine-rich RNA-binding protein 8 | Down |
| AT4G39940 | APK2 | Adenylyl-sulfate kinase 2, chloroplastic | Down |
| AT4G39960 | | Molecular chaperone Hsp40/DnaJ family protein | Down |
| AT4G39980 | DHS1 | Phospho-2-dehydro-3-deoxyheptonate aldolase 1, chloroplastic | Down |
| AT5G02760 | | Probable protein phosphatase 2C 67 | Up |
| AT5G02840 | RVE4 | Protein REVEILLE 4 | Up |
| AT5G05460 | ENGASE1 | Cytosolic endo-beta-N-acetylglucosaminidase 1 | Up |
| AT5G06530 | ABCG22 | ABC transporter G family member 22 | Up |
| AT5G08130 | BIM1 | Transcription factor BIM1 | Up |
| AT5G11970 | | Protein of unknown function (DUF3511) | Up |
| AT5G18470 | | Curculin-like (mannose-binding) lectin family protein | Up |
| AT5G20230 | BCB | Blue copper protein | Up |
| AT5G20630 | GER3 | Germin-like protein subfamily 3 member 3 | Down |
| AT5G20740 | | Plant invertase/pectin methylesterase inhibitor superfamily protein | Down |
| AT5G22000 | RHF2A | E3 ubiquitin-protein ligase RHF2A | Up |
| AT5G25250 | FLOT1 | Flotillin-like protein 1 | Up |
| AT5G25265 | | unknown protein | Up |
| AT5G25350 | EBF2 | EIN3-binding F-box protein 2 | Up |
| AT5G25910 | AtRLP52 | receptor like protein 52 | Up |
| AT5G26570 | GWD3 | Phosphoglucan, water dikinase, chloroplastic | Down |
| AT5G42530 | | unknown protein | Up |
| AT5G44820 | | Nucleotide-diphospho-sugar transferase family protein | Up |
| AT5G45500 | | RNI-like superfamily protein | Up |
| AT5G45550 | MOB1A | MOB kinase activator-like 1A | Up |
| AT5G45650 | | subtilase family protein | Down |
| AT5G46330 | FLS2 | LRR receptor-like serine/threonine-protein kinase FLS2 | Up |
| AT5G47240 | NUDT8 | Nudix hydrolase 8 | Down |
| AT5G47370 | HAT2 | Homeobox-leucine zipper protein HAT2 | Up |
| AT5G47560 | TDT | Tonoplast dicarboxylate transporter | Up |
| AT5G48657 | | defense protein-related | Up |
| AT5G49570 | PNG1 | Peptide-N(4)-(N-acetyl-beta-glucosaminyl)asparagine amidase | Up |
| AT5G52320 | CYP96A4 | cytochrome P450, family 96, subfamily A, polypeptide 4 | Down |
| AT5G52900 | MAKR6 | Probable membrane-associated kinase regulator 6 | Up |
| AT5G53550 | YSL3 | Metal-nicotianamine transporter YSL3 | Up |

| | | | |
|---|---|---|---|
| AT5G54960 | PDC2 | Pyruvate decarboxylase 2 | Down |
| AT5G55460 | | Bifunctional inhibitor/lipid-transfer protein/seed storage 2S albumin superfamily protein | Up |
| AT5G57110 | ACA8 | Calcium-transporting ATPase 8, plasma membrane-type | Down |
| AT5G59080 | | unknown protein | Down |
| AT5G59680 | | Probable LRR receptor-like serine/threonine-protein kinase At5g59680 | Up |
| AT5G60100 | APRR3 | Two-component response regulator-like APRR3 | Down |
| AT5G60540 | PDX2 | Probable pyridoxal 5'-phosphate synthase subunit PDX2 | Down |
| AT5G60950 | COBL5 | COBRA-like protein 5 | Up |
| AT5G61210 | SNAP33 | SNAP25 homologous protein SNAP33 | Up |
| AT5G62280 | | Protein of unknown function (DUF1442) | Up |
| AT5G62360 | | Plant invertase/pectin methylesterase inhibitor superfamily protein | Down |
| AT5G63980 | SAL1 | Inositol monophosphatase family protein | Down |
| AT5G66200 | ARO2 | armadillo repeat only 2 | Up |
| AT5G66640 | DAR3 | Protein DA1-related 3 | Up |
| AT5G67340 | PUB2 | U-box domain-containing protein 2 | Up |
