# Supplementary material for: Enhancement of Arabidopsis growth characteristics using genome interrogation with artificial transcription factors
Source: PLoS One. 2017 Mar 30;12(3):e0174236. doi: 10.1371/journal.pone.0174236 (PMC5373528; doi:10.1371/journal.pone.0174236)
Supplement: S9 Table — (PDF) [file pone.0174236.s014.pdf]

S9 Table. Overview of significantly enriched GO categories ($p < 0.05$) found for the 146 upregulated (**Up**) and 93 downregulated (**Down**) DEGs compared to the wild type Col-0 that are shared in the RNA sequencing data sets of the two larger 3F-VP16 transgenic lines, VP16-02-003 and VP16-005-014.

**Up**						
**GO biological process**	**GO term**	**Total number of genes in GO term**	**Number of genes**	**Expected value**	**Fold Enrichment**	**P-value**
response to salicylic acid	GO:0009751	157	8	0.86	9.35	5.88E-03
response to organic cyclic compound	GO:0014070	268	11	1.46	7.53	6.57E-04
regulation of response to stimulus	GO:0048583	396	11	2.16	5.1	2.66E-02
cellular response to hormone stimulus	GO:0032870	625	15	3.4	4.41	4.07E-03
hormone-mediated signaling pathway	GO:0009755	587	14	3.2	4.38	9.74E-03
cellular response to endogenous stimulus	GO:0071495	630	15	3.43	4.37	4.48E-03
response to lipid	GO:0033993	546	13	2.97	4.37	2.20E-02
cellular response to organic substance	GO:0071310	728	16	3.97	4.03	5.59E-03
response to acid chemical	GO:0001101	860	18	4.68	3.84	2.54E-03
cellular response to chemical stimulus	GO:0070887	827	17	4.51	3.77	6.51E-03
response to organic substance	GO:0010033	1453	29	7.92	3.66	2.63E-06
response to hormone	GO:0009725	1190	23	6.48	3.55	2.99E-04
response to endogenous stimulus	GO:0009719	1270	23	6.92	3.32	9.28E-04
response to oxygen-containing compound	GO:1901700	1122	20	6.11	3.27	7.15E-03
signal transduction	GO:0007165	1377	23	7.5	3.07	3.66E-03
single organism signaling	GO:0044700	1404	23	7.65	3.01	5.05E-03
signaling	GO:0023052	1405	23	7.65	3.01	5.11E-03
multi-organism process	GO:0051704	1279	20	6.97	2.87	4.76E-02
response to chemical	GO:0042221	2053	32	11.18	2.86	1.17E-04
cell communication	GO:0007154	1560	24	8.5	2.82	8.68E-03
cellular response to stimulus	GO:0051716	2000	29	10.89	2.66	2.42E-03
protein modification process	GO:0036211	1718	24	9.36	2.56	4.27E-02
cellular protein modification process	GO:0006464	1718	24	9.36	2.56	4.27E-02
response to stimulus	GO:0050896	4612	52	25.12	2.07	1.66E-04
biological regulation	GO:0065007	4649	46	25.33	1.82	4.70E-02
cellular process	GO:0009987	9225	76	50.25	1.51	2.13E-02
**Down**						
**GO biological process**	**GO term**	**Total number of genes in GO term**	**Number of genes**	**Expected value**	**Fold Enrichment**	**P-value**
defense response by callose deposition in cell wall	GO:0052544	15	3	0.05	58.82	4.24E-02
defense response by cell wall thickening	GO:0052482	15	3	0.05	58.82	4.24E-02

glucosinolate biosynthetic process	GO:0019761	27	4	0.09	43.57	5.32E-03
glycosinolate biosynthetic process	GO:0019758	27	4	0.09	43.57	5.32E-03
sulfur compound biosynthetic process	GO:0044272	82	8	0.28	28.69	1.09E-06
response to wounding	GO:0009611	173	12	0.59	20.4	2.19E-09
response to jasmonic acid	GO:0009753	166	8	0.56	14.17	2.44E-04
sulfur compound metabolic process	GO:0006790	258	10	0.88	11.4	4.54E-05
response to endogenous stimulus	GO:0009719	1270	16	4.32	3.71	1.20E-02
response to external stimulus	GO:0009605	1246	15	4.24	3.54	4.14E-02
response to stress	GO:0006950	2664	27	9.06	2.98	3.04E-04
response to stimulus	GO:0050896	4612	35	15.68	2.23	2.81E-03
